# Supplementary material for: Dosing and Re-Administration of Lentiviral Vector for In Vivo Gene Therapy in Rhesus Monkeys and ADA-Deficient Mice
Source: Mol Ther Methods Clin Dev. 2019 Nov 16;16:78–93. doi: 10.1016/j.omtm.2019.11.004 (PMC6909201; doi:10.1016/j.omtm.2019.11.004)
Supplement: Document S1. Figures S1–S4 and Tables S1–S5 [file mmc1.pdf]

OMTM, Volume *16*

Supplemental Information

Dosing and Re-Administration of Lentiviral Vector for *In Vivo* Gene Therapy in Rhesus Monkeys and ADA-Deficient Mice

Denise A. Carbonaro-Sarracino, Alice F. Tarantal, C. Chang I. Lee, Michael L. Kaufman, Stephen Wandro, Xiangyang Jin, Michele Martinez, Danielle N. Clark, Krista Chun, Colin Koziol, Cinnamon L. Hardee, Xiaoyan Wang, and Donald B. Kohn

Supplemental Figures and Tables

Figure S1. Vector Maps. All vectors are self-inactivating (SIN) lentiviral vectors (LV), with the U3, R, and U5 regions of the LTR shown with the SIN deletion indicated by the "X". HIV-1 or $SIV_{mac1A11}$ packaging region (Ψ), 5' portion of the gag gene (gag), rev-responsive element (rre), and central polypurine tract (cPPT) are indicated. **(a)** Vectors used for the murine studies. All are VSV-pseudotyped SIN HIV-1 (CCL or CSO) vectors with either the MNDU3 enhancer/promoter (MND-ADA) or the short version of the elongation factor-1 promoter (EFS-ADA) with or without a 5' A2 ubiquitous chromatin opening element (UCOE) driving human *Ada cDNA* or eGFP expression. **(b)** Vectors used for the rhesus monkey studies. All are VSV-pseudotyped SIN $SIV_{mac1A11}$ LV (CL20) with the MSCV viral enhancer/promoter. The SIV-ADA expresses human *Ada cDNA*. SIV-FX LV carries the non-expressed gene sequences derived from PhiX174 and the dominant-negative human RevM10 gene. SIV-NeoNT LV carries the bacterial transposon neomycin-resistance gene with the translational start codon eliminated (NeoNT).

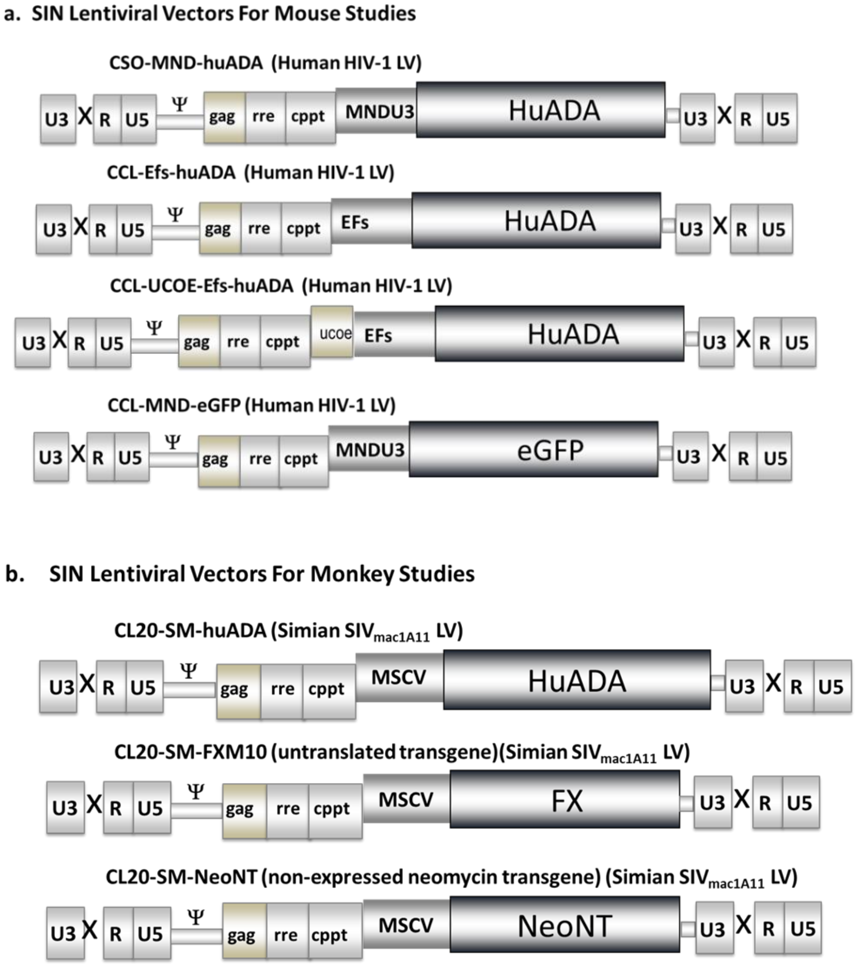

Figure S2: Immunophenotyping and absolute cell counts in mice systemically administered LV expressing human ADA. Immunophenotyping of cells by flow cytometry in thymus and spleen at time of analysis. *Ada+/-* mice untreated control (n= 9), *Ada-/-* mice treated with ERT only (n= 10), *Ada-/-* mice treated as adults (n=14), *Ada-/-* mice treated as adults twice within three days (n= 5), *Ada-/-* mice treated as neonates (n= 15), *Ada+/-* mice treated as neonates (n=13), *Ada-/-* mice untreated (16 days old) (n=6). **(a)** Absolute spleen and thymus cell counts (x10e7), total organ cell count (viable and non-viable). **(b-c)** Thymocyte subpopulations: single positive cells (SP CD4+ and SP CD8+), double positive cells (DP CD4+CD8+) and double negative cells (DN CD4-CD8-). (b) percentage in gate and (c) absolute counts (proportion x total organ cell count). **(d-e)** Splenocyte subsets: T cells (CD45+CD3+, CD3+CD4+, CD3+CD8+) and B cells (CD45+CD19+). **(d)** percentage in gate and **(e)** absolute counts (proportion x total organ cell count). Shown geometric means ±95% CI.

a.

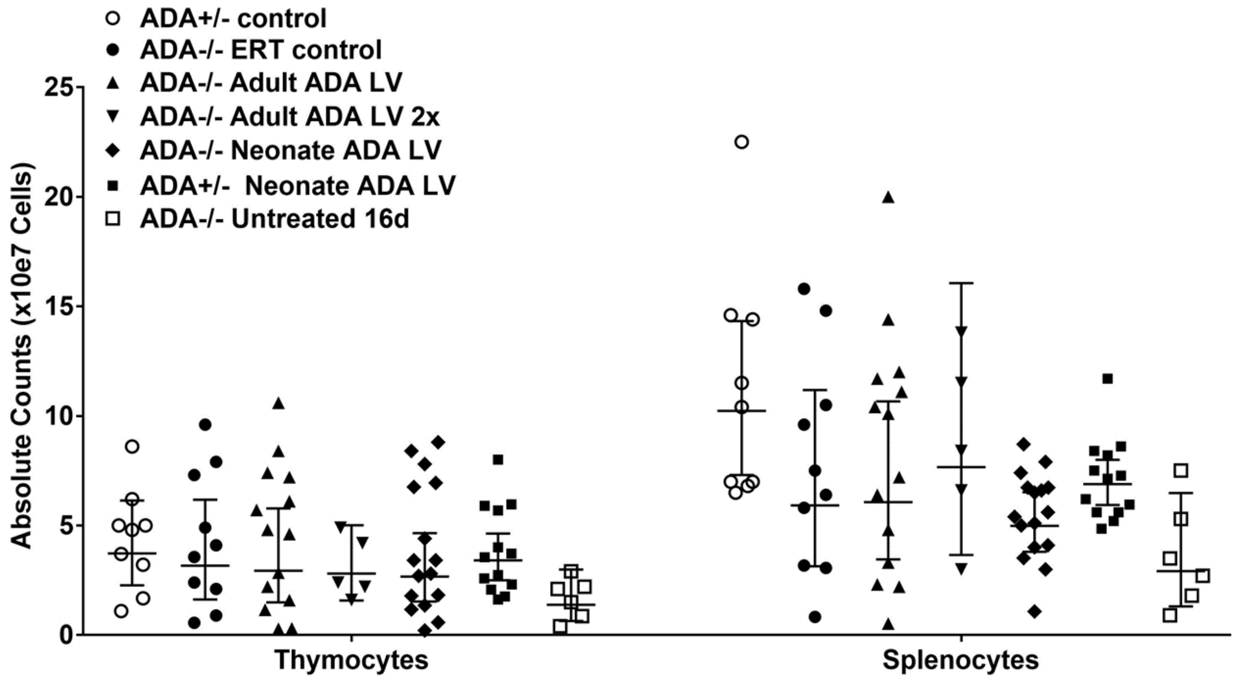

b.

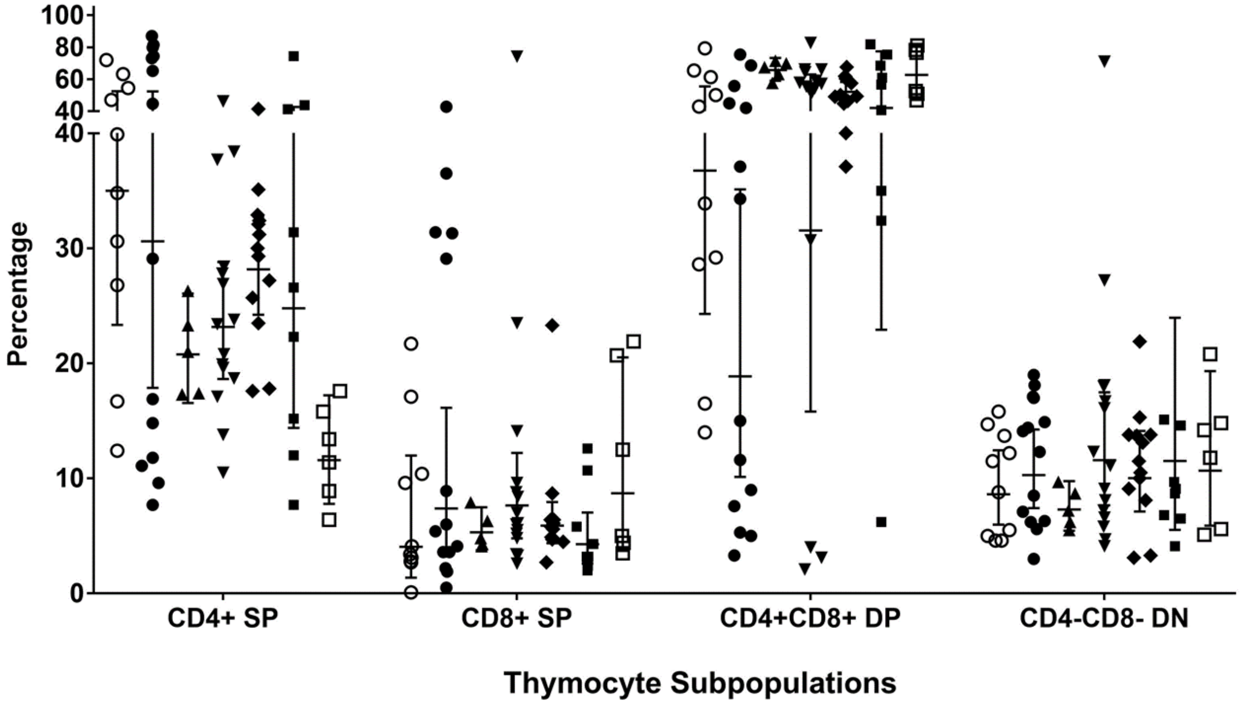
Percentage
100
80
60
40
40
30
20
10
0
CD4+ SP
CD8+ SP
CD4+CD8+ DP
CD4-CD8- DN
Thymocyte Subpopulations

c.

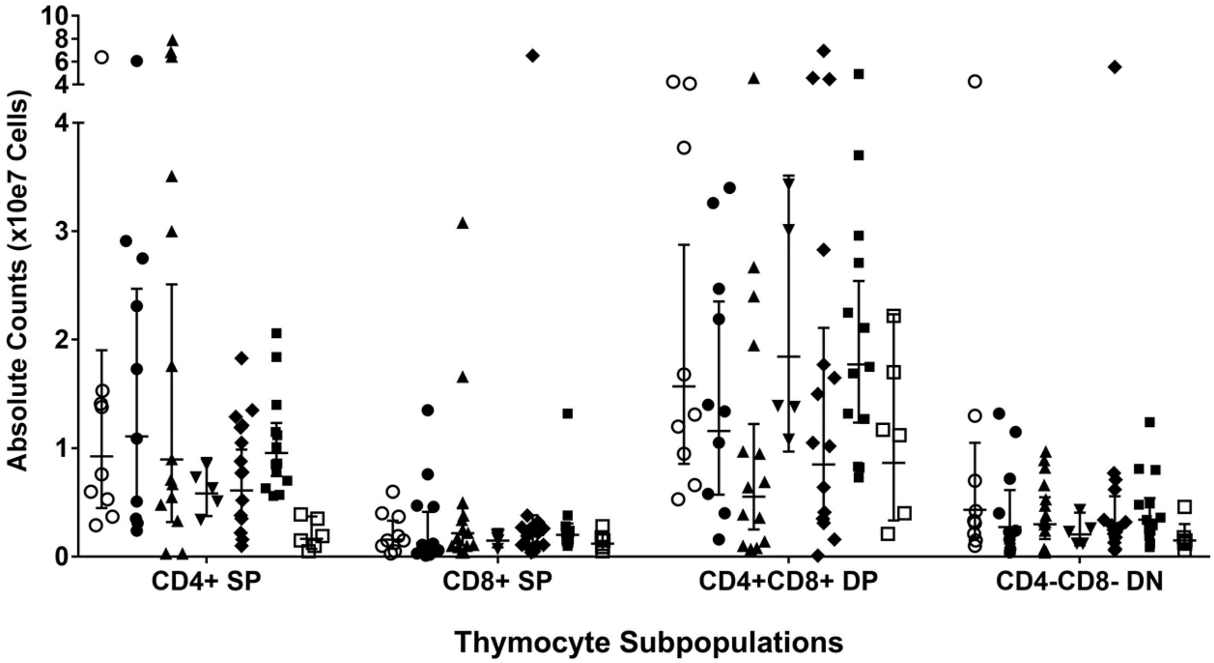
Absolute Counts (x10e7 Cells)
10
8
6
4
4
3
2
1
0
CD4+ SP
CD8+ SP
CD4+CD8+ DP
CD4-CD8- DN
Thymocyte Subpopulations

d.

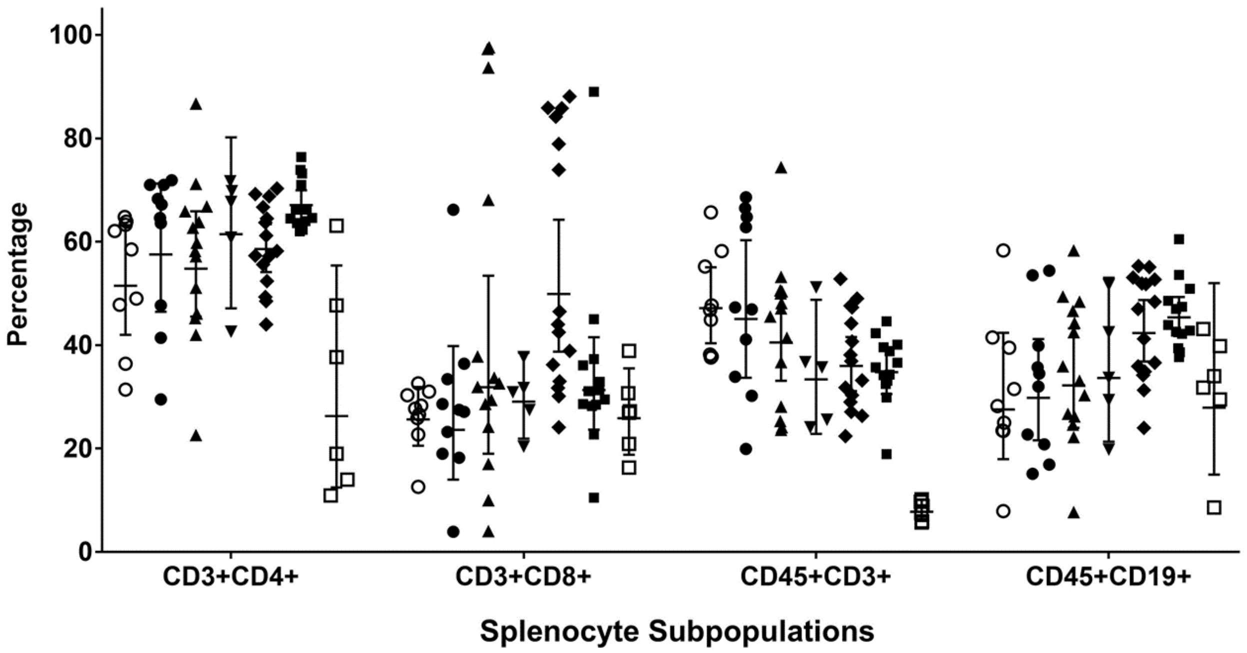

100
80
60
40
20
0
Percentage
CD3+CD4+
CD3+CD8+
CD45+CD3+
CD45+CD19+
Splenocyte Subpopulations

e.

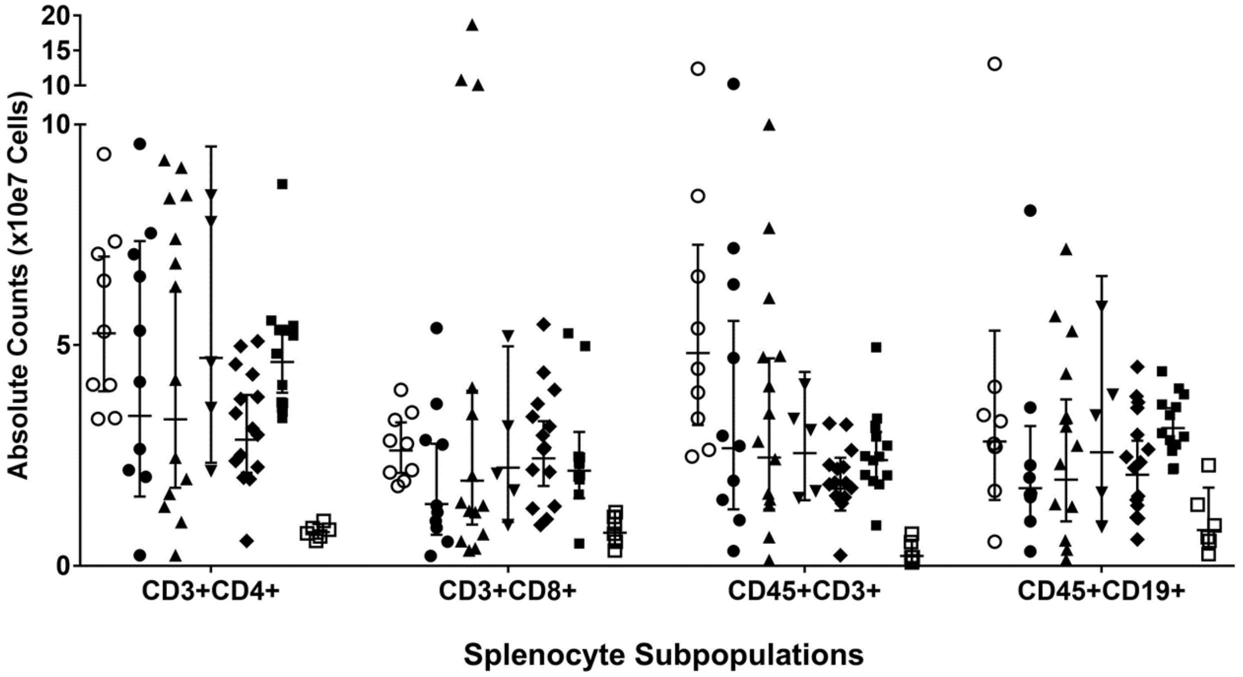

20
15
10
10
5
0
Absolute Counts (x10e7 Cells)
CD3+CD4+
CD3+CD8+
CD45+CD3+
CD45+CD19+
Splenocyte Subpopulations

Figure S3: Anti-vector response in HEK 293T lysates. ELISA using HEK 293T packaging cell line lysates (1:500) as the capture antigen compared to concentrated LV vector (1:1000 dilution). 293T cells were either transfected with the packaging plasmid 8.9 (expressing gag, pol, and rev), the env plasmid pHIT123 (VSV-G), or both.

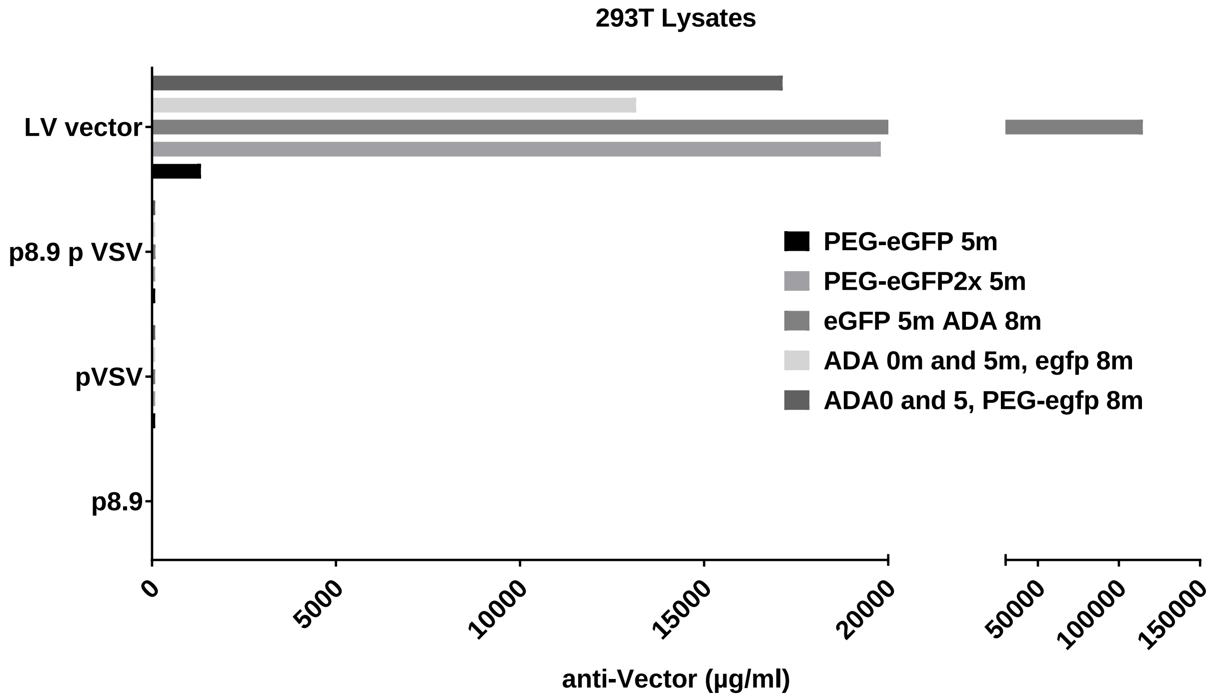

Figure S4. Spleen and liver VCN and ADA enzyme activity after systemic administration of MND-ADA LV, EFS-ADA LV or UCOE-EFS-ADA LV. Spleen (a-c) and Liver (d-f); (a and d) VCN was determined with ddPCR using primers and probes to human ADA as described previously (16); (b and e) ADA enzyme assay performed using the Diazyme ADA enzyme assay as described previously (16) (Unit=One unit of ADA is defined as the amount of ADA that generates one µmole of inosine from adenosine per mg per min at 37°C); (c and e) ADA Activity normalized for VCN (Units/VCN).

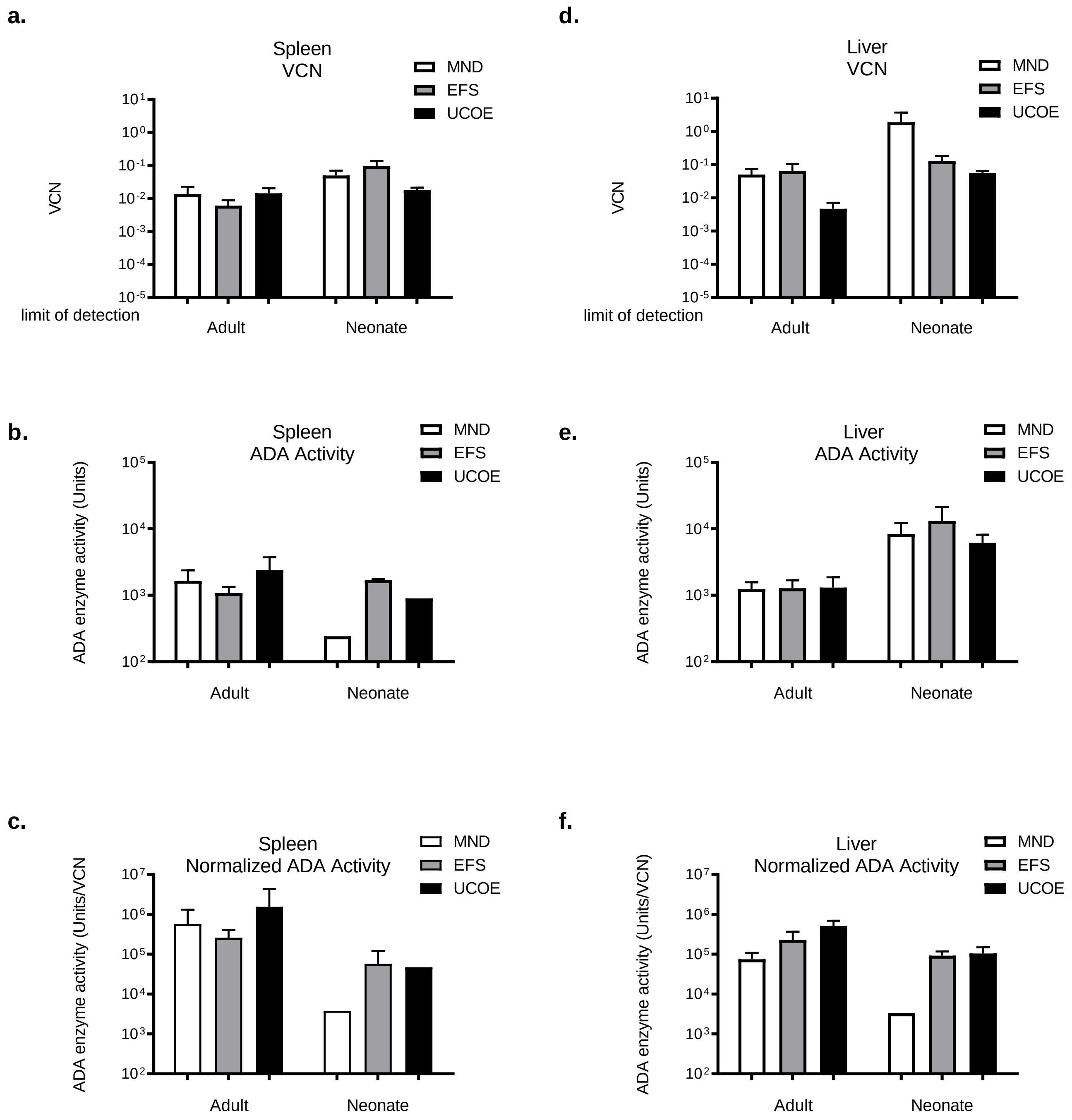

Table S1. Characterization of eGFP LV and PEG-eGFP LV

Vector Name	Fold Concentrated	PEG ELISA (ng/ml) 2500X dilution	PEG (ng/ml)	Titer (TU/ml)
Unconcentrated eGFP vector	0	0	0	2.1x10e6
Partially concentrated vector (PCV)	55	0	0	5.0x10e7
eGFP PCV	27.5	0	0	1.6x10e7
PEG-eGFP PCV	27.5	60	150,000	1.8x10e7
eGFP LV	1,000	0	0	6.6x10e8
PEG-eGFP LV	1,000	110	275,000	8.8x10e8

N/A=not available, PEG=polyethylene glycol, ELISA=enzyme linked immunosorbent assay

Table S2. Study design of rhesus monkey studies

Study number	Group (N)	Lentiviral Vectors (N)	Parameters
1<br>(n= 6)	Dose response at birth	SIV-ADA LV<br>1.0x10e10 TU/kg (3)<br>1.0x10e11 TU/kg (3)	Blood collection 0, 1, 3, 7, 14 and 30 days then every other month post-administration. Tissue harvest at 12 months of age.
2<br>(n= 6)	Dose response at 4 months	SIV-ADA LV<br>3.7x10e9 TU/kg (3)<br>3.7x10e10 TU/kg (3)	Blood collection 0, 1, 3, 7, 14 and 30 days then monthly post-administration. Tissue harvest at 10 months of age.
3<br>(n= 6)	Vector at 1 month<br>Vector at 4 months<br>Vector at 1 and 4 months	SIV-FX LF (2)<br>SIV-NeoNT LV (2)<br>SIV-FX LV+ SIV-NeoNT LV (2)	Blood collection 0, 1, 3, 7, 14 and 30 days then monthly post-administration. Tissue harvest at 7 months of age.
4<br>(n= 6)	Vector at 3 months<br>Vector at 6 months<br>Vector at 3 and 6 months	SIV-FX LV (2)<br>SIV-PEG-NeoNT LV (2)<br>SIV-FX LV+ SIV-PEG-NeoNT (2)	Blood collection 0, 1, 3, 7, 14 and 30 days then monthly post-administration. Tissue harvest at 10 months of age.

*All newborns were delivered by cesarean section and raised in the nursery for postnatal studies.
SIV=simian immunodeficiency virus ($SIV_{mac1A11}$ nonpathogenic clone); VSV=vesicular stomatitis virus glycoprotein; PEG=polyethylene glycol;
FX=Phi X-174 bacteriophage DNA; NeoNT=non-expressed neomycin resistance gene
**Tissue harvests included: brain (cerebrum, cerebellum), lung (all lobes), trachea, esophagus, heart, aorta, pericardium, thymus, spleen, liver (all lobes), lymph nodes (axillary, inguinal, tracheobronchial, mesenteric), pancreas, adrenals, kidneys, reproductive tract (gonads, uterus or seminal vesicles and prostate), gastrointestinal tract (stomach, duodenum, jejunum, ileum, colon), muscular component of the diaphragm, omentum, peritoneum (muscular component of body wall), skin, muscle, and bone marrow.

Table S3. Repeat Administration of SIV vectors: Liver marking and anti-vector response at study endpoint

Monkey Pairs	Vector at 1st time point	Vector at 2nd time point	Liver marking with FX-LV	Liver marking with NeoNT-LV	Baseline Anti-vector IgG	Anti-vector IgG 3 m after 1st time point	Anti-vector IgG 6 m after 1st time point
1 and 2	FX @ 1 m	N/A	Yes	N/A	No	Yes	Yes
3 and 4	N/A	NeoNT@ 4 m	N/A	Yes	No	No	Yes
5 and 6	FX @ 1 m	NeoNT @ 4 m	Yes	No	No	Yes	Yes
7 and 8	N/A	PEG-NeoNT @ 6 m	N/A	Yes	No	No	Yes
9 and 10	FX @ 3 m	NeoNT @ 6 m	Yes	No	No	Yes	Yes
11 and 12	FX @ 3 m	PEG-NeoNT @ 6 m	Yes	No	No	Yes	Yes

N/A=Not applicable, FX=non-expressed FXM10 gene, NeoNT=non-expressed neomycin resistance gene, m=month or months

Table S4. Characterization of the FX LV, NeoNT LV, and the PEG-NeoNT LV

Vector Name	Fold Concentrated	PEG ELISA (ng/ml) 5000X dilution	PEG (ng/ml)	Titer (TU/ml)
NeoNT unconcentrated vector	0	N/A	N/A	3.5x10e7
Partially concentrated vector	120	0	0	N/A
NeoNT PCV	26	0	0	N/A
PEG-NeoNT PCV	60	26	130,000	N/A
Flow through during concentration	N/A	21	105,000	N/A
FX unconcentrated vector	0	N/A	N/A	1.1x10e7
FX LV	1,000	N/A	N/A	2.4x10e9
NeoNT LV	1,000	0	0	1.6x10e10
PEG-NeoNT LV	1,000	28	140,000	9.6x10e9

N/A=not available, PEG=polyethylene glycol, PCV=partially concentrated vector, FX=non-expressed gene based on phi174, NeoNT=non-expressed neomycin resistance gene

Table S5. Response to 293T lysates (Mice)

			Bound Antigen<br>Transfected HEK 293T cell lysate<br>(1:500)			LV vector (1:1,000)
Time of Vector Administration			p8.9	pVSV	p8.9 p VSV	Avg anti-vector IgG response
0 months	5 months	8 months	µg/µL	µg/µL	µg/µL	µg/µL
	PEG-eGFP LV		7.8	90.0	89.0	1,330.0
	PEG-eGFP LV 2x		7.6	87.0	88.0	19,800.0
	eGFP-LV	ADA LV	8.0	88.0	91.0	114,666.7
ADA LV	ADA LV	eGFP LV	8.0	88.0	89.0	13,150.0
ADA LV	ADA LV	PEG-eGFP LV	7.6	87.0	88.0	17,133.3

Reactivity to lysates of 293T cells transfected with either the p8.9 (gag, pol, rev, tat), pHit123 (VSV-G), or both.
Lysates were used as bound antigen diluted 1:500 in carbonate binding buffer.
Concentrated LV was diluted 1:1000 in carbonate binding buffer.
Sera from treated *Ada+/-* mice, tested are pooled plasma n= 3-4 from each treatment arm.
