# Supplementary material for: Dosing and Re-Administration of Lentiviral Vector for In Vivo Gene Therapy in Rhesus Monkeys and ADA-Deficient Mice
Source: Mol Ther Methods Clin Dev. 2019 Nov 16;16:78–93. doi: 10.1016/j.omtm.2019.11.004 (PMC6909201; doi:10.1016/j.omtm.2019.11.004)
Supplement: Document S2. Article plus Supplemental Information [file mmc2.pdf]

Molecular Therapy
Methods & Clinical Development
Original Article

Dosing and Re-Administration of Lentiviral Vector for *In Vivo* Gene Therapy in Rhesus Monkeys and ADA-Deficient Mice

Denise A. Carbonaro-Sarracino,[1] Alice F. Tarantal,[2,3] C. Chang I. Lee,[2,3] Michael L. Kaufman,[1] Stephen Wandro,[1] Xiangyang Jin,[1] Michele Martinez,[2,3] Danielle N. Clark,[1] Krista Chun,[1] Colin Koziol,[1] Cinnamon L. Hardee,[1] Xiaoyan Wang,[4] and Donald B. Kohn[1,5,6]

[1]Department of Microbiology, Immunology, and Molecular Genetics, University of California, Los Angeles, Los Angeles, CA 90095, USA; [2]Center for Fetal Monkey Gene Transfer for Heart, Lung, and Blood Diseases, University of California, Davis, Davis, CA 95616, USA; [3]Departments of Pediatrics and Cell Biology and Human Anatomy, School of Medicine, and California National Primate Research Center, University of California, Davis, Davis, CA 95616, USA; [4]Department of General Internal Medicine and Health Services Research, University of California, Los Angeles, Los Angeles, CA 90095, USA; [5]Department of Pediatrics, David Geffen School of Medicine, University of California, Los Angeles, Los Angeles, CA 90095, USA; [6]The Eli & Edythe Broad Center for Stem Cells and Regenerative Medicine, University of California, Los Angeles, Los Angeles, CA 90095, USA

Adenosine deaminase (ADA)-deficient mice and healthy rhesus monkeys were studied to determine the impact of age at treatment, vector dosage, dosing schedule, repeat administration, biodistribution, and immunogenicity after systemic delivery of lentiviral vectors (LVs). In *Ada*$^{-/-}$ mice, neonatal treatment resulted in broad vector marking across all tissues analyzed, whereas adult treatment resulted in marking restricted to the liver, spleen, and bone marrow. Intravenous administration to infant rhesus monkeys also resulted in dose-dependent marking in the liver, spleen, and bone marrow. Using an ELISA to monitor anti-vector antibody development, *Ada*$^{-/-}$ neonatal mice did not produce an antibody response, whereas *Ada*$^{-/-}$ adult mice produced a strong antibody response to vector administration. In mice and monkeys with repeat administration of LV, a strong anti-vector antibody response was shown in response to the second LV administration, which resulted in LV inactivation. Three separate doses administered to immune competent mice resulted in acute toxicity. Pegylation of the vesicular stomatitis virus G protein (VSV-G)-enveloped LVs showed a less robust anti-vector response but did not prevent the inactivation of the second LV administration. These studies identify important factors to consider related to age and timing of administration when implementing systemic delivery of LVs as a potential therapeutic agent.

INTRODUCTION

Lentiviral vectors (LVs) have been described as an efficient delivery vehicle suitable for direct injection and gene delivery *in vivo*, owing mostly to the ability to achieve gene transfer in non-dividing cells.[1] Since these initial studies, systemic delivery of LVs has been considered for a number of monogenic diseases, including the coagulation deficiencies hemophilia A[2] and hemophilia B,[3] inborn errors of metabolism such as lysosomal storage disorders MPS1,[4] MPS3a,[5] MPS3b,[6] bilirubin-UDP-glucuronosyltransferase deficiency (Crigler-Najjar),[7] Pompe disease,[8] and adenosine deaminase (ADA) deficiency,[9] among others. In many instances, systemic delivery of LVs was first investigated as a neonatal approach in murine and other animal models to prevent or ameliorate disease pathology.[10]

ADA deficiency is an inborn error of metabolism that results in the accumulation of adenosine and deoxyadenosine metabolites resulting from absent or aberrant *Ada gene* expression. Patients are most notably stricken with severe combined immunodeficiency (SCID), as normal lymphocyte development is severely impaired by the accumulation of these metabolites.[11] Infants typically present with severe and persistent infections characterized by a failure-to-thrive and profound lymphopenia. In addition to SCID, affected ADA-deficient individuals may also have hepatic, renal, pulmonary, skeletal, and/or neurological pathology associated with the accumulation of metabolites.[12]

ADA-deficient patients with a matched sibling donor can be treated soon after diagnosis with hematopoietic stem cell transplantation (HSCT). If no suitable donor is available, a patient may be stabilized with enzyme replacement therapy (ERT) bovine ADA conjugated to polyethylene glycol (PEG-ADA) (ADA-GEN, Leadiant Biotechnologies, Gaithersburg, MD, USA). ERT can substantially increase lymphocyte counts and provide some immune reconstitution; long-term use, however, has been associated with waning immune cell numbers and function.[13] More recently, ERT has been accepted as

Received 6 August 2019; accepted 4 November 2019;
https://doi.org/10.1016/j.omtm.2019.11.004.

Correspondence: Donald B. Kohn, Department of Microbiology, Immunology, and Molecular Genetics, University of California, Los Angeles, Los Angeles, CA 90095, USA.
E-mail: dkohn1@mednet.ucla.edu

 © 2019 The Authors.
This is an open access article under the CC BY license (http://creativecommons.org/licenses/by/4.0/).

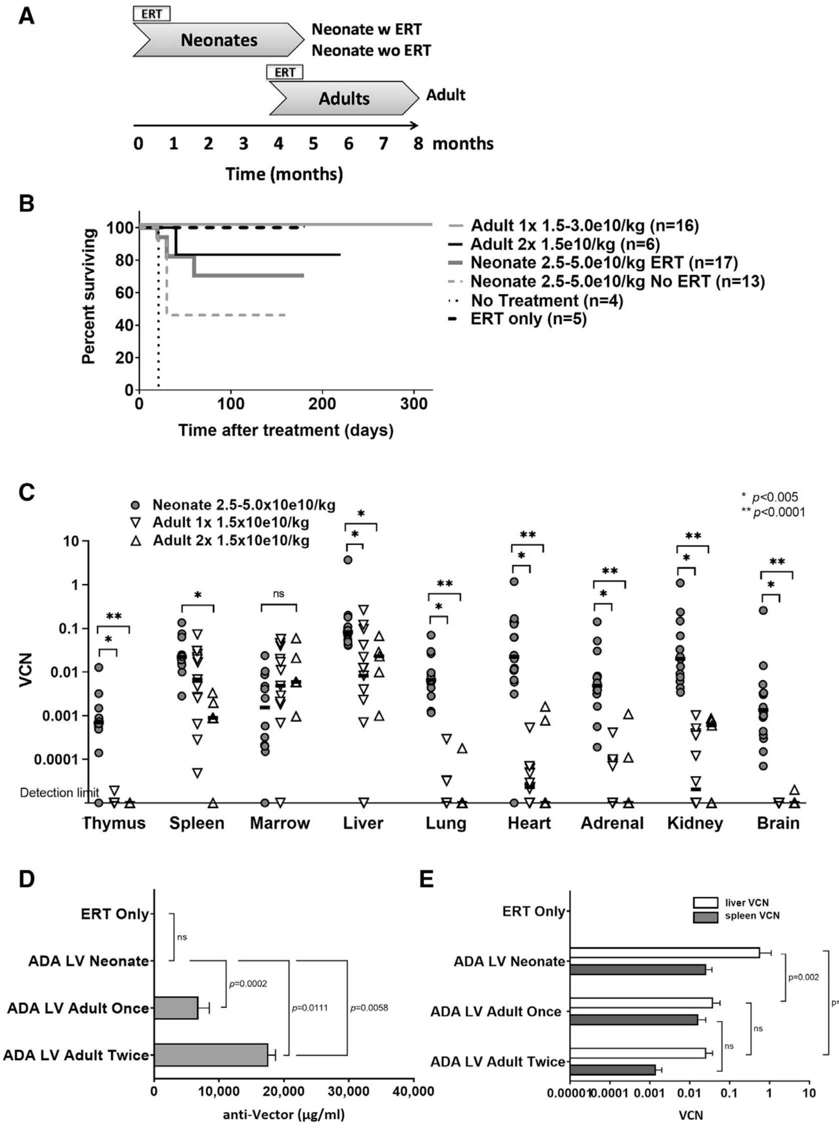

Figure 1. Survival, Biodistribution, and Immunogenicity in *Ada*$^{-/-}$ Mice

(A) Schematic of vector administration and analysis (arrow) timeline. Controls not depicted. (B) Kaplan-Meier survival curves. (C) Biodistribution and quantitation of vector sequences (VCNs) in tissue. Lines represent the median VCN in each tissue: Neonate (n = 16), Adult 1× (N = 15), and Adult 2× (n = 5). (D) Mean (±SEM) concentration of anti-vector antibody in plasma (μg/mL): ERT (n = 5), Neonate (N = 7), Adult 1× (N = 12), Adult 2× (n = 5). (E) Mean (±SEM) VCN measured in liver (light bar) and spleen (dark bar): ERT (n = 5), Neonate (n = 7), Adult 1× (n = 12), Adult 2× (n = 5).

an important bridge to a more durable HSC treatment.[14] In recent clinical trials, many patients have been successfully treated with autologous HSCs *ex vivo* gene therapy using retroviral (gamma and lentiviral) gene-corrected CD34+ hematopoietic stem and progenitor cells (HSCT GT).[15]

For patients where a stem cell therapy may not be an option, including older patients with ADA deficiency with partial ADA expression associated with late/adult onset, enzyme replacement by *in vivo Ada* gene delivery could provide an alternative therapeutic approach. In prior studies, we reported that a single injection of ADA-expressing LVs could rescue ADA-deficient (*Ada*$^{-/-}$) neonatal mice, which would typically succumb to adenosine and deoxyadenosine accumulation in the lungs by postnatal day 21 (~3 weeks).[9] However, it seems unlikely that most patients with late onset ADA-deficiency will be identified and treated within the neonatal period. Thus, further studies in animal models beyond the neonatal period are essential for understanding the effect of age on treatment outcome, as well as the dosing schedule (including repeat administration), and the potential to elicit an immune response. In these studies, we used the ADA-deficient mouse model and healthy young rhesus monkeys to address these questions. Importantly, the findings described herein provide valuable insights for preclinical studies that include systemic administration of LVs for the treatment of other monogenic diseases.

RESULTS

Dosing Schedule and Biodistribution in *Ada*$^{-/-}$ Mice

In previous studies, *Ada*$^{-/-}$ mice were rescued in a dose-dependent manner by systemic intravenous administration of a lentiviral vector (LV)-expressing human ADA, surviving past 3 weeks without further treatment.[9] *Ada*$^{-/-}$ neonates treated with 5.0 × 10e9 TU/kg (1.0 × 10e7 TU/neonate) did not survive past day 30, while those treated with a 10-fold higher dose of 5.0 × 10e10 TU/kg (1.0 × 10e8 TU/neonate) survived with good immune reconstitution and resolution of the lethal pulmonary insufficiency.[9] In another related study, biodistribution analyses demonstrated differences in the amount of LVs detected in *Ada*$^{-/-}$ mice treated as newborns (at birth) compared to healthy infant rhesus monkeys treated at 1 month of age where no vector was detected in the rhesus thymus or brain.[16] However, it was not clear whether the differences observed were species-specific, developmental age-specific, or disease-specific.

In these studies, neonatal *Ada*$^{-/-}$ and adult *Ada*$^{-/-}$ mice were treated with an intravenous injection of LV expressing the human *Ada* gene (ADA LV) to assess the effects of age on survival and LV biodistribution (Figure 1A; Figure S1). Litters of *Ada*$^{-/-}$ and *Ada*$^{+/-}$

mice were treated as neonates with each pup receiving a dose of 2.5–5.0 × 10e10 TU/kg of ADA LV ("Neonate groups"). Some litters were treated with supplemental polyethylene glycol (PEG)-ADA ERT for the first month post treatment (Neonate ERT) and others received no supplemental ERT post-treatment (Neonate No ERT) (Table 1). *Ada*$^{-/-}$ mice treated at 4 months of age comprised the Adult groups and either received a single dose of 1.5-3 × 10e10/kg (Adult 1×) or two doses of 1.5–10e10/kg within 3 days (Adult 2×). The Adult group were administered PEG-ADA ERT from birth until the time of LV treatment at 4 months of age and then for 1 month post-LV treatment.

Survival of *Ada*$^{-/-}$ mice in the Neonate group with ERT was 70.6%. When compared to untreated *Ada*$^{-/-}$ mice, the probability of survival was higher ($p = 0.0493$) (Figure 1B). Survival of *Ada*$^{-/-}$ mice in the Neonate group without ERT was 46.2% and was not significantly different when compared to untreated *Ada*$^{-/-}$ mice ($p = 0.8802$). Survival of *Ada*$^{-/-}$ mice in the Adult group was 100% when administered a single dose and was 83.3% when treated with two doses. Compared to untreated *Ada*$^{-/-}$ mice, the probability of survival was significantly higher in *Ada*$^{-/-}$ Adult mice with a single dose (Adult 1×; $p = 0.0001$) or two doses (Adult 2×; $p = 0.0016$).

As observed in our prior studies,[9,16] *Ada*$^{-/-}$ mice in the Neonate group, treated with a single ADA LV administration, showed the highest vector copy number (VCN) in liver (0.337 ± 0.241 copies/cell) (Figure 1C). Similarly, marking was also highest in liver when mice were treated as Adults once (Adult 1×, 0.039 ± 0.0187 copies/cell) or twice within 3 days (Adult 2×, 0.026 ± 0.0114 copies/cell). In the Adult group treated once, results in tissue VCN were significantly lower (10- to 1,000-fold) in thymus ($p < 0.0001$), liver ($p = 0.002$), lung ($p < 0.0001$), heart ($p < 0.0001$), adrenal glands ($p < 0.0001$), kidneys ($p < 0.0001$), and brain ($p < 0.0001$) compared to the Neonate group. Similar results were observed in the Adult group treated twice compared to those in the Neonate group treated once, with tissue VCN significantly lower in thymus ($p = 0.004$), spleen ($p = 0.003$), liver ($p = 0.005$), lung ($p = 0.003$), heart ($p = 0.003$), adrenal glands ($p = 0.002$), kidneys ($p = 0.001$), and brain ($p = 0.001$). The only tissue not significantly different was bone marrow in mice in the Adult group treated once or twice ($p = 0.165$). Due to vector concentration and delivery volume constraints, mice in the Adult group received approximately one-third (Adult 1×) to one-half (Adult 2×) the neonatal dose, which could explain the decreased marking in these tissues; however, in many tissues the difference in marking far exceeded the difference in dose by 10- to 1,000-fold.

In previous studies, we reported that *Ada*$^{-/-}$ mice surviving beyond 3 weeks after treatment with intravenous LV without supplemental ERT had sufficient ADA enzyme activity to reconstitute the lymphocyte compartment.[9,17] Likewise, in this study there were no differences across the treatment modalities in lymphocyte cell percentages or absolute numbers (Figure S2).

Anti-Vector Responses in *Ada*$^{-/-}$ Mice

To determine the potential for producing anti-vector antibodies and inactivating subsequent administration of vesicular stomatitis virus G protein (VSV-G)-pseudotyped LV, we developed an ELISA using concentrated stocks of LV as the capture antigen for the detection of anti-vector antibodies (immunoglobulin G, IgG) in the plasma of treated mice.[18] The relative concentration of anti-vector antibodies was quantified by comparing the anti-vector IgG concentration in the sample to the signal detected in the standard curve of serially diluted primary mouse anti-VSV-G IgG.

To determine whether *Ada*$^{-/-}$ mice treated with systemic ADA LV could mount an adaptive immune response to initial and subsequent vector administration, we measured the concentrations of anti-vector antibodies in plasma samples at the time of analysis (Figure 1D). In *Ada*$^{-/-}$ mice in the Neonate group, the mean (±SEM) anti-vector IgG concentration at 8 months post treatment was slightly above background (29 ± 18 μg/μL) and not significantly different than *Ada*$^{-/-}$ mice treated only with ERT ($p = 0.1931$) (Figure 1D). In contrast, in *Ada*$^{-/-}$ mice treated in the Adult group, the anti-vector concentration at 4 months post treatment was 200-fold higher (6,842 ± 1,653 μg/μL) after one vector dose ($p = 0.0002$) and over 500-fold higher (17,612 ± 1,089 μg/μL) after two doses given 3 days apart ($p = 0.0055$) when compared to *Ada*$^{-/-}$ mice in the Neonate group. Interestingly, *Ada*$^{-/-}$ mice in the Adult group treated twice within 3 days had a significantly stronger response compared to those treated once ($p = 0.0111$) without significant changes in liver ($p = 0.430$) or spleen ($p = 0.0760$) VCN (Figure 1E). A time course performed on treated *Ada*$^{-/-}$ mice in the Adult group revealed that a strong anti-vector response developed within the first month after treatment and was stable from month 1 to month 4 post treatment (Table 2).

Anti-vector Response to PEG-Modified LVs and Repeat Administration in *Ada*$^{+/-}$ Mice

We investigated the use of LVs modified with conjugated PEG to reduce the potential for vector inactivation as previously reported.[19] PEG molecules were conjugated to lysine residues within the VSV-G at the diafiltration step during vector concentration.[20]

To control for the pegylation process, we split one batch of partially concentrated enhanced GFP (eGFP) LV (27.5-fold) into two fractions, where one fraction underwent the pegylation process and the other fraction remained unmodified (Table S1). The PEG-eGFP LV titer was 8.8 × 10e8 TU/mL and the unmodified eGFP LV titer was 6.6 × 10e8 TU/mL, indicating pegylation did not diminish the viral titer. Using direct competitive ELISA, the PEG molecules conjugated to each LV was quantified. The unmodified eGFP LV had no detectable PEG, whereas the PEG-eGFP LV had 275,000 ng/mL of PEG.

To delineate the role of the adaptive immune response in the persistence of liver marking in immune-competent mice, we compared *Ada*$^{+/-}$ littermates treated with LV as Neonates in the same manner as their *Ada*$^{-/-}$ littermates. Additionally, older *Ada*$^{+/-}$ mice were

Table 1. Viral Vector, Titer, and Dose in Mouse and Rhesus Monkey Studies

Vector Used in Studies	Short Name	Transgene Expressed	Titer (TU/mL)	Dose (TU/kg)	Age of Treatment	PEG Modified
**Mouse Studies (Figures 1, 2, and 3)**						
HIV-MNDU3-ADA/VSV	(MND-ADA-LV)	yes	8.0 × 10e9	5.0 × 10e10	birth	no
HIV-EFS-ADA/VSV	(EFS-ADA-LV)	yes	7.0 × 10e9	2.5 × 10e10	birth	no
HIV-UCOE-EFS-ADA/VSV	(UCOE-EFS-ADA-LV)	yes	1.0 x10e10	2.5-5.0 × 10e10	birth	no
HIV-MNDU3-ADA/VSV	(MND-ADA-LV)	yes	8.0 × 10e9	1.5 × 10e10	4–5 months	no
HIV-EFS-ADA/VSV	(EFS-ADA-LV)	yes	7.0 × 10e9	1.5 × 10e10	4–5 months	no
HIV-UCOE-EFS-ADA/VSV	(UCOE-EFS-ADA-LV)	yes	1.0 × 10e10	1.5 × 10e10	4–5 months	no
HIV-MNDU3-eGFP/VSV	(eGFP-LV)	yes	8.8 × 10e8	1.7 × 10e8	4–5 months	no
HIV-MNDU3-eGFP/VSV-PEG	(PEG-eGFP-LV)	yes	6.6 × 10e8	1.7 × 10e8	4–5 months	yes
**Rhesus Monkey Study 1 and 2 (Figure 4)**						
SIV-MNDU3-ADA/VSV	(SIV-ADA-LV)	yes	3.6 × 10e9	1.0 × 10e10	birth	no
SIV-MNDU3-ADA/VSV	(SIV-ADA-LV)	yes	2.0 × 10e10	1.0 × 10e11	birth	no
SIV-MNDU3-ADA/VSV	(SIV-ADA-LV)	yes	3.7 × 10e10	3.7 × 10e9	4 months	no
SIV-MNDU3-ADA/VSV	(SIV-ADA-LV)	yes	3.7 × 10e10	3.7 × 10e10	4 months	no
**Rhesus Monkey Study 3 (Figures 5 and Table S3)**						
SIV-FXM10/VSV	(FX-LV)	no	2.0 × 10e10	2.0 × 10e9	1 month	no
SIV-NeoNT/VSV	(NeoNT-LV)	no	1.0 × 10e10	2.0 × 10e9	4 months	no
**Rhesus Monkey Study 4 (Figures 6 and Table S3)**						
SIV-FXM10/VSV	(FX-LV)	no	2.9 × 10e9	2.0 × 10e9	3 months	no
SIV-NeoNT/VSV	(NeoNT-LV)	no	1.6 × 10e10	2.0 × 10e9	6 months	no
SIV-NeoNT/VSV-PEG	(PEG-NeoNT-LV)	no	9.6 × 10e9	2.0 × 10e9	6 months	yes

VSV, vesicular stomatitis virus glycoprotein; HIV-1, human immunodeficiency virus 1; SIV, simian immunodeficiency virus; FX, Phi X-174 bacteriophage DNA; NeoNT, non-expressed neomycin resistance gene; PEG, polyethylene glycol; TU, transducing units.

treated as Adults once at 5 months of age (Figure 2A). *Ada*$^{+/-}$ mice are phenotypically normal and do not succumb if no treatment is given. All *Ada*$^{+/-}$ mice treated as Neonates or Adults with unmodified LV or PEG-modified LV survived after treatment (Figure 2B).

Immune-competent *Ada*$^{+/-}$ mice treated once as Neonates with the ADA LV had similar mean liver VCN (0.128 ± 0.066 copies) and anti-vector IgG concentration (275 ± 193 μg/mL) when compared to the mean liver VCN (0.084 ± 0.056 copies; p = 0.5912) and anti-vector IgG concentration (1,137 ± 2.5 μg/mL; p = 0.1065) in *Ada*$^{+/-}$ mice treated once with the ADA LV as Adults at 5 months (Figures 2C and 2D). *Ada*$^{+/-}$ mice treated as Adults with the eGFP LV received a lower dose due to low titer and volume constraints, compared to those *Ada*$^{+/-}$ mice treated as Neonates with the ADA LV. However, despite the higher dose, those *Ada*$^{+/-}$ mice treated as Neonates with ADA LV had significantly lower anti-vector IgG concentration compared to *Ada*$^{+/-}$ mice treated as Adults at 5 months with the eGFP LV (p = 0.0084), PEG-eGFP LV (p = 0.0029), PEG-eGFP LV twice within 3 days (p = 0.0221), or PEG-eGFP LV twice at 5 and 8 months of age (p = 0.0141) (Figure 2C). In contrast, there were no significant differences in either the anti-vector IgG concentration or liver VCN in *Ada*$^{+/-}$ mice treated at 5 months with a single administration of the ADA LV, the unmodified eGFP LV, or the PEG-eGFP LV, suggesting no increased immunogenicity of the eGFP LVs. Only those *Ada*$^{+/-}$ mice treated with the PEG-eGFP LV at 5 and 8 months had a significantly higher anti-vector IgG concentration compared to those treated once with the eGFP LV (p = 0.0369) or the PEG-eGFP LV (p = 0.0222). Additionally, the mean VCN at the time of analysis was 10- to 100-fold lower in mice treated with the PEG-eGFP LV twice at 5 and 8 months compared with those mice treated at 5 months once with the PEG-eGFP LV (p = 0.0518) or twice within 3 days with the PEG-eGFP LV (p = 0.0722); however, these differences were not statistically significant. Taken together, these results suggest that pegylation of the eGFP LV did not provide protection against an anti-vector response.

To study the potential for LV inactivation, we treated mice at different time points with two different vectors each carrying distinct transgenes, allowing for detection of each vector (Figure 3A). As seen in the previous study with the same type of vector, administration of two different LVs was well tolerated, with all mice surviving to the end of the study (Figure 3B). The anti-vector IgG mean concentration

Table 2. Anti-Vector IgG Concentration at 1, 2, and 4 Months after Treatment of Mice with a Single Dose of LV

					Anti-Vector Antibody after Treatment (μg/mL)		
Mouse ID	Genotype	LV Treatment	Age of Treatment	Dose	1 Month	2 Months	4 Months
1573	$Ada^{+/-}$	eGFP	adult	1.65 × 10e8/kg	144	144	144
1583	$Ada^{+/-}$	eGFP	adult	1.65 × 10e8/kg	674	680	658
1586	$Ada^{+/-}$	eGFP	adult	1.65 × 10e8/kg	2,440	2,340	2,380
1572	$Ada^{+/-}$	PEG-eGFP	adult	1.65 × 10e8/kg	2,380	2,380	2,300
1579	$Ada^{+/-}$	PEG-eGFP	adult	1.65 × 10e8/kg	690	686	676
1585	$Ada^{+/-}$	PEG-eGFP	adult	1.65 × 10e8/kg	6,740	6,820	6,760
1588	$Ada^{+/-}$	PEG-eGFP	adult	1.65 × 10e8/kg	2,320	2,360	2,360
1543	$Ada^{-/-}$	EFS-ADA	adult	3.0 × 10e10/kg	1,760	1,792	1,766
1548	$Ada^{-/-}$	EFS-ADA	adult	3.0 × 10e10/kg	1,808	1,888	1,826
1552	$Ada^{-/-}$	EFS-ADA	adult	3.0 × 10e10/kg	1,360	1,340	1,320
1549	$Ada^{-/-}$	MND-ADA	adult	3.0 × 10e10/kg	2,560	2,540	2,560
1555	$Ada^{-/-}$	MND-ADA	adult	3.0 × 10e10/kg	N/A	158	138
1556	$Ada^{-/-}$	MND-ADA	adult	3.0 × 10e10/kg	1,876	1,868	1,836
1569	$Ada^{-/-}$	MND-ADA	adult	3.0 × 10e10/kg	17,700	17,360	17,660
1544	$Ada^{-/-}$	UCOE-EFS-ADA	adult	3.0 × 10e10/kg	1,776	1,828	1,880
1554	$Ada^{-/-}$	UCOE-EFS-ADA	adult	3.0 × 10e10/kg	2,560	2,556	2,570
1571	$Ada^{-/-}$	UCOE-EFS-ADA	adult	3.0 × 10e10/kg	1,900	1,818	1,836
1576	$Ada^{-/-}$	UCOE-EFS-ADA	adult	3.0 × 10e10/kg	2,576	2,551	2,555

ID, Identification; LV, lentiviral vector; N/A, not available.

after $Ada^{+/-}$ mice treated as Neonates (0 months) with the ADA LV and at 8 months with the eGFP LV was not different when compared to mice treated with the ADA LV at birth and the PEG-eGFP LV at 8 months (Figure 3C). When Adult mice were initially treated with the unmodified eGFP LV at 5 months, followed by the ADA LV at 8 months, the mean anti-vector IgG concentration was increased 11-fold (114,667 ± 667 μg/mL) compared to mice first treated with the PEG-eGFP LV at 5 months and the ADA LV at 8 months (9,733 ± 333 μg/mL); the difference was not statistically significant (p = 0.0722). When the ADA LV was administered first, both vectors were detected and there were no differences in the ADA LV liver VCN or in the subsequent the eGFP LV liver VCN, regardless of whether the eGFP LV was pegylated or not (Figure 3D). Remarkably, when the ADA LV was administered as the second vector after treatment with either the eGFP LV or the PEG-eGFP LV, no ADA LV was detected in liver.

To further test re-administration and the potential for inactivation, we gave $Ada^{+/-}$ mice previously treated with an ADA LV as neonates a "booster" of the same ADA LV at 5 months and with the eGFP LV (n = 5) or PEG-eGFP LV (n = 5) at 8 months. Unexpectedly, three administrations of the LV over 8 months reduced the probability of survival to 60% in both treatment arms, with loss of mice within 30–60 min of receiving the third LV administration (Figure 3B). Those that survived the third injection were inactive post injection but recovered within 1 h and survived to the study endpoint. In the surviving mice, there were no differences in anti-vector IgG concentration in mice treated with the ADA LV at birth and at 5 months and the eGFP LV at 8 months (17,466 ± 466 μg/mL) compared to mice treated with the ADA LV at birth and at 5 months and the PEG-eGFP LV at 8 months (17,133 ± 2018 μg/mL) (p = 0.6579). Likewise, there were no differences (p = 0.1052) in the mean liver VCN with the ADA LV administered at birth and 5 months, nor with the unmodified eGFP LV (0.003 ± 0.001 ADA copies, 0.238 ± 0.208 eGFP copies) or PEG-eGFP LV (0.063 ± 0.05 ADA copies, 0.028 ± 0.017 eGFP copies) administered at 8 months (Figures 3C and 3D).

Dosing Schedule and Biodistribution in Rhesus Monkeys

In parallel studies, we determined the biodistribution of simian immunodeficiency virus $(SIV)_{mac1A11}$-based LVs in rhesus monkeys when administered intravenously at birth (study 1) or 4 months of age (study 2) (Table 1; Table S2). Previously, we compared the biodistribution of HIV-1 LVs and SIV LVs in rhesus neonates and found the biodistribution to be similar despite the well-described TRIM5α-mediated host restriction observed in nonhuman primate HSCs to HIV-1 viral vector integration.[16] We used the species-specific SIV LVs pseudotyped with the VSV-G envelope in these studies. The expression cassette contained the MND enhancer/promoter driving expression of human *Ada cDNA* (Figure S1)[9]. All rhesus monkeys had birth weights and growth trajectories within the normative range (data not shown). Blood samples were collected for complete blood counts (CBCs) and chemistry panels, all of which were within the anticipated range for rhesus

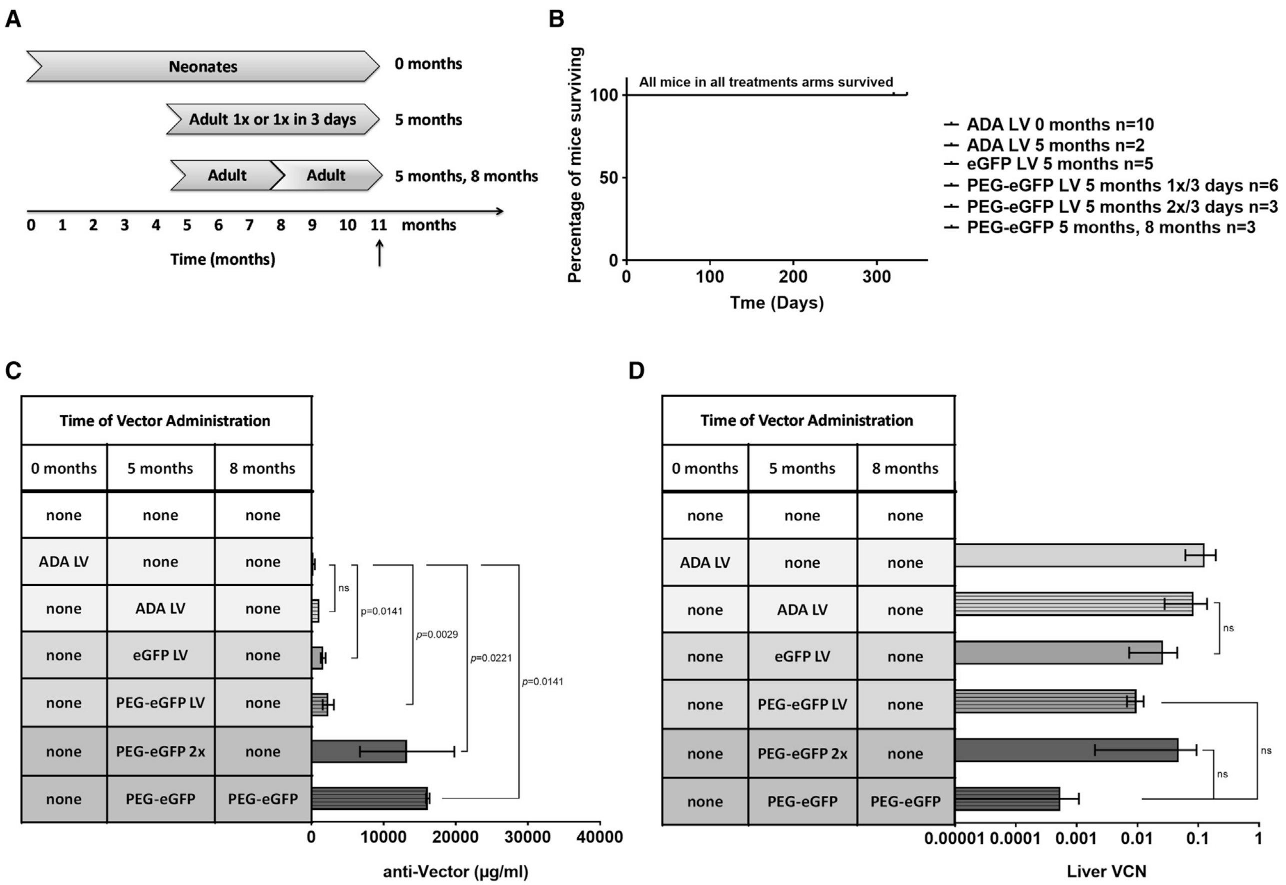

Figure 2. Immunogenicity of Unmodified and PEGylated LVs in Immune-Competent *Ada*$^{+/-}$ Mice

(A) Schematic of study design. (B) Kaplan-Meier survival curves. (C) Mean (±SEM) concentration of anti-vector antibodies (µg/mL). (D) Mean (±SEM) liver VCNs.

monkeys in this age group (data not shown). There were no adverse events observed in any of the animals throughout the study and to the endpoint (~1 year of age).

To model biodistribution in young animals, we treated rhesus monkeys at birth (study 1) or at 4 months of age (study 2) in dose-response studies. In study 1, rhesus monkeys were administered the SIV-ADA LV at birth at one of two dose levels, 1.0 × 10e10 TU/kg (n = 3) or 1 × 10e11 TU/kg (n = 3) with tissue harvests performed at 12 months of age (12 months post transfer). VCN was dose-dependent with vector sequences detected in liver and adrenal glands at both dose levels and in lung, heart, kidneys, omentum, spleen, and bone marrow only at the higher dose (Figure 4A). In study 2, rhesus monkeys were administered the LV at 4 months of age at one of two dose levels, 3.7 × 10e9 TU/kg (n = 3) or 3.7 × 10e10 TU/kg (n = 3) with tissue harvests performed at 10 months of age (6 months post transfer). In general, the VCN increased in liver and spleen with increased dose and vector was detected in the heart, adrenal glands, muscle, lymph nodes, and bone marrow only at the higher dose (Figure 4B). Overall, liver VCN ranged from 0.0008 to 0.015 and was similar at higher and lower doses studied when comparing rhesus monkeys treated at birth or 4 months of age. Furthermore, in rhesus monkeys treated at 4 months of age, marking was less than in those treated at birth when comparing VCN in lung, heart, and adrenal glands, whereas it was higher in spleen, lymph nodes, and bone marrow. It is possible the difference in marking may be due to the maturation of the immune system (between birth and 4 months of age).

Re-administration of LV in Immune-Competent Rhesus Monkeys

To investigate the potential for inactivation of vector upon re-administration, we administered to rhesus monkeys the SIV LV carrying the non-expressed transgene, FXM10 (FX LV) or the non-expressed neomycin resistance gene (NeoNT LV) (Figure 5; Table S3). All LVs were administered intravenously at a dose of 2.0 × 10e9 TU/kg (Table 1).

In study 3, rhesus monkeys #1 and #2 were treated with the FX LV at 1 month of age (Figures 5A and 5B) and rhesus monkeys #3 and #4 were treated with the NeoNT LV at 4 months of age (Figures 5C and 5D). Rhesus monkeys #5 and #6 were treated with the FX LV

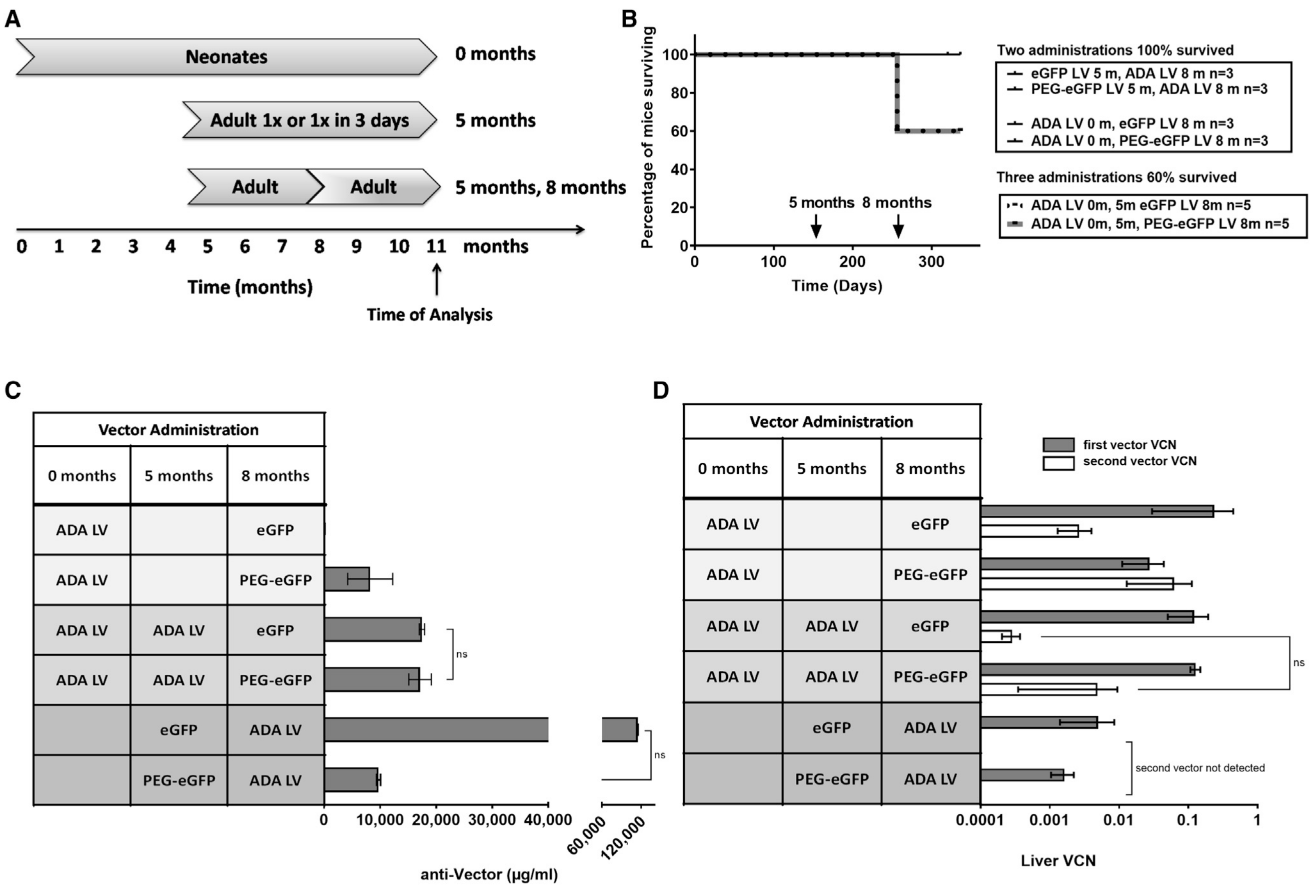

Figure 3. Repeat Administration of Unmodified and PEGylated LVs in Immune-Competent *Ada*$^{+/-}$ Mice

(A) Schematic of study design. Dark arrows represent the first vector administered and light arrows represent the second vector administered. (B) Kaplan-Meier survival. (C) Mean (±SEM) concentration of anti-vector antibodies (μg/mL). (D) Mean (±SEM) liver VCN of each transgene: first-administered LV transgene (gray bar) and the second-administered LV transgene (black bar).

at 1 month of age and the NeoNT LV at 4 months of age (Figures 5E and 5F). Tissue harvests were performed at 7 months of age (6 months post vector administration when treated at 1 month of age or 3 months post transfer when last vector administration was at 4 months of age). Liver VCN was similar in rhesus monkeys treated with FX LV at 1 month only or with the NeoNT LV at 4 months only. However, in animals treated with the FX LV at 1 month and with the NeoNT LV at 4 months, there was no detectable NeoNT LV copies in liver or in any tissue analyzed (data not shown). Anti-vector IgG was detected within 2 to 3 months after the first exposure to LV, with IgG concentrations in rhesus monkeys administered both the FX LV at 1 month and NeoNT LV at 4 months similar to those detected in monkeys treated with only a single LV. These results suggest the anti-vector response after one exposure was sufficient for complete inactivation of the second LV administered.

In study 4, we set out to determine whether a pegylated LV would prevent the inactivation of the second-administered LV. Rhesus monkeys were treated with the FX LV at 3 months of age and with either the unmodified NeoNT LV or with the NeoNT LV that was modified by pegylation (PEG-NeoNT LV) at 6 months of age (Figure 6; Table S3). The NeoNT LV titer was 1.6 × 10e10 TU/mL and the PEG-NeoNT LV titer was 9.6 × 10e9 TU/mL. The NeoNT LV had 0 ng/mL PEG molecules, while the PEG-NeoNT LV had 275,000 ng/mL PEG molecules (Table S4). All LVs were administered intravenously at a dose of 2.0 × 10e9 TU/kg (Table 1).

Rhesus monkeys #7 and #8 were treated with the PEG-NeoNT LV at 6 months of age (Figures 6A and 6B), #9 and #10 were treated first with the FX LV at 3 months of age followed by the unmodified NeoNT LV at 6 months of age (Figures 6C and 6D), and #11 and #12 were first treated with the FX LV at 3 months of age followed by the PEG-NeoNT LV at 6 months of age (Figures 6E and 6F). Tissue harvests were performed at 10 months of age (4 months after the last vector administration). These studies resulted in liver marking similar to the marking observed in study 3 with the unmodified NeoNT, suggesting that the PEG-NeoNT LV was able to transduce the liver.

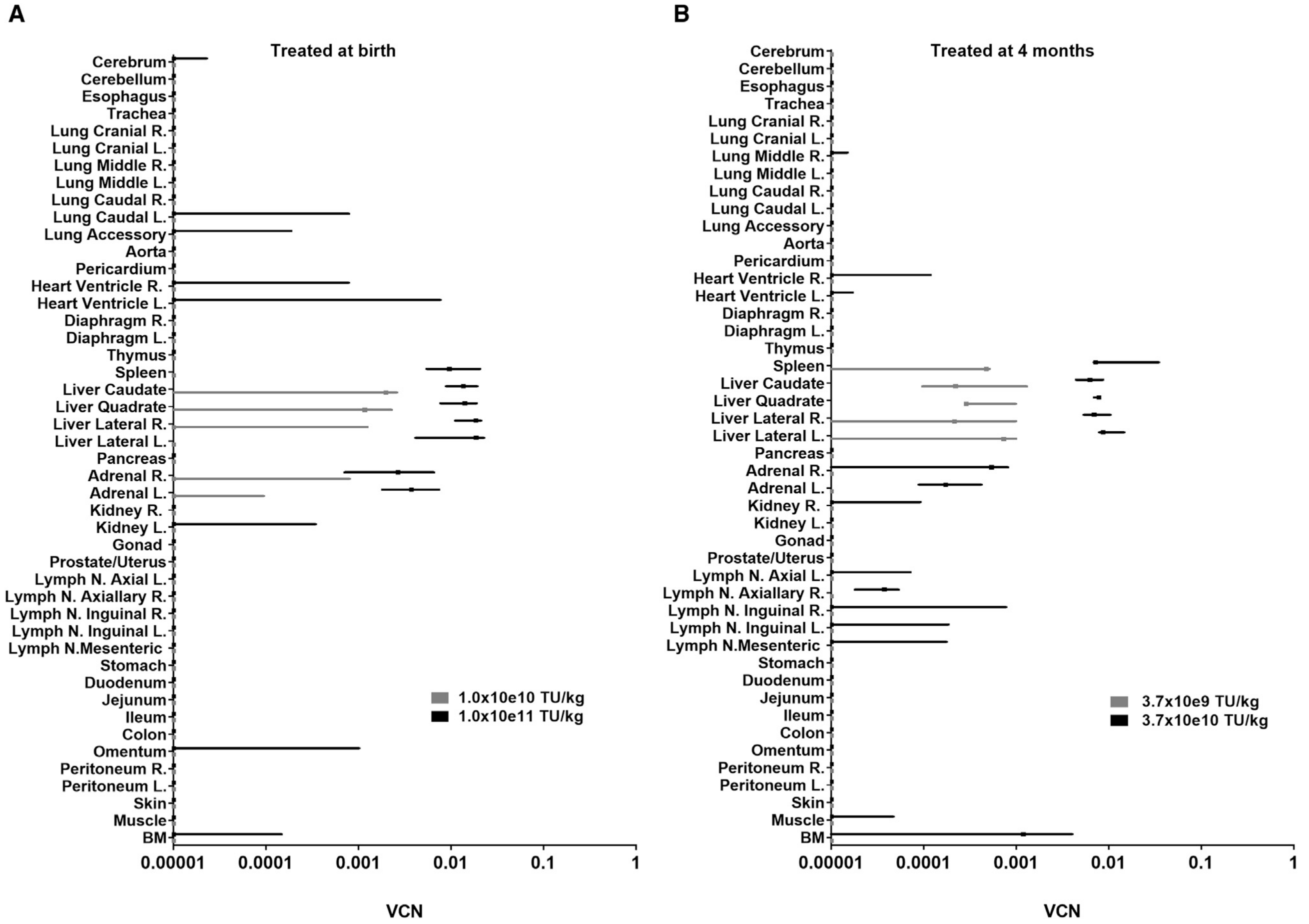

Figure 4. Biodistribution in Rhesus Monkeys: Dose Response after LV Administration at Birth or 4 Months of Age

(A) Biodistribution of LVs after rhesus monkey newborns were treated at birth (study 1) with an intravenous injection of the SIV-ADA LV at 1.0 × 10e10 TU/kg (n = 3) or 1.0 × 10e11 TU/kg (n = 3). (B) Biodistribution of LVs after rhesus monkeys were treated at 4 months of age (study 2) with an intravenous injection of the SIV-ADA LV at 3.7 × 10e9 TU/kg (n = 3) or 3.7 × 10e11 TU/kg (n = 3). Data are plotted as 10th–90th percentile whisker plots with 50th percentile denoted (median).

In the rhesus monkeys treated with both vectors, only the first vector administered (FX LV) was detected, whereas none of the monkeys had evidence of marking with the subsequent administration of the NeoNT LV or the PEG-NeoNT LV. Further, there was no difference in the anti-vector response after injection of the first LV. These results suggest that pegylation of LV was unable to prevent inactivation of the second LV administered and that the response to vector administration at first exposure was sufficient to inactivate the second injection.

Further Characterization of the Anti-Vector Response in Mouse and Rhesus Monkey Samples

The specificity of the anti-vector response was further characterized in terms of cross-reactivity to different antigen capture LVs. LVs evaluated in the ELISA had different transgenes, vector backbones, and pseudotypes. Plasma samples from treated mice and monkeys tested included LVs carrying expressed transgenes (ADA, eGFP) and non-expressed transgenes (FX and NeoNT). In all cases, the magnitude of the antibody response against LVs with expressed and non-expressed transgenes was not significantly different, nor were the responses specific to the LV and transgene to which the animal was exposed. Plasma from treated rhesus monkeys was equivalently reactive to SIV LV and HIV LV capture antigens, even though treated animals were only exposed to SIV LV (data not shown). Similarly, plasma from LV-treated mice was equivalently reactive to SIV LV and HIV LV capture antigens, although mice were only exposed to HIV LV (data not shown), suggesting that the response is not specific for capsid or gag proteins p27 or p24, respectively. Comparing plasma samples from mice treated with LV pseudotyped with VSV-G were equivalently reactive to LV pseudotyped with murine amphotropic leukemia virus envelope, murine ecotropic Moloney leukemia virus envelope, Gibbon-Ape virus envelope, or RD114 endogenous feline virus envelope. In all mouse plasma tested, the results were similar, indicating the response was not VSV-G specific (data not shown). Taken together, these data suggest that the response is not LV specific.

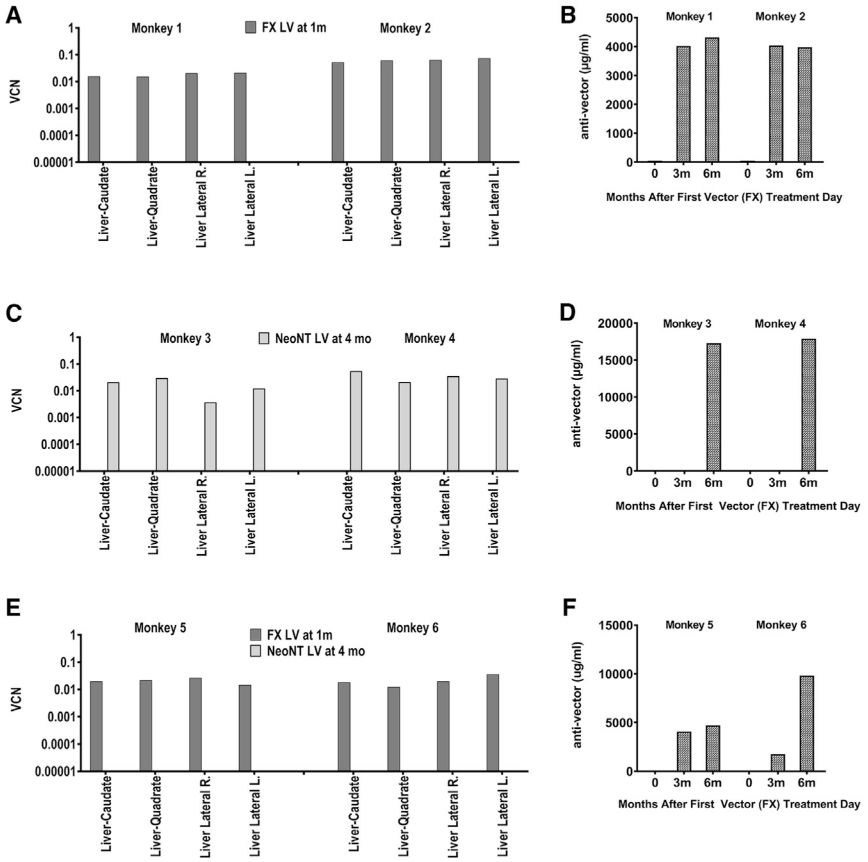

Figure 5. Repeat Administration of VSV-Pseudotyped LVs with Non-expressed Transgenes in Immune-Competent Rhesus Monkeys

Liver VCN (A, C, and E) and the concentration of anti-vector antibody detected in plasma (μg/mL) at 0 (pre), 3, and 6 months after the first vector treatment (B, D, and F) are shown for each pair of treated monkeys. (A and B) Rhesus monkeys #1 and #2 were administered the FX LV at 1 month of age. (C and D) Rhesus monkeys #3 and #4 were administered the NeoNT LV at 4 months of age. (E and F) Rhesus monkeys #5 and #6 were administered both the FX LV at 1 month of age and the NeoNT LV at 4 months of age.

Additional ELISAs were performed using lysates from HEK293T cells as capture antigens. The packaging cell lines were transfected with the VSV-G expression plasmid alone, with the packaging plasmid (gag-pol) alone, or with both the VSV-G plasmid and the gag-pol plasmid. Plasma samples from several test conditions were pooled as the supply of murine plasma samples was limited. There was no response to the capture antigen from lysates of 293T cells transfected with the packaging plasmid alone, but the concentration of antibody increased 10-fold above background with lysates of 293T cells transfected with the VSV-G plasmid alone or together with the packaging plasmid. However, all responses were 1,000- to 100,000-fold lower than the response to the concentrated viral vector preparation depending on the test condition (Table S5; Figure S3).

DISCUSSION

The initial focus of our studies with systemic LV administration centered on the specific disease model of ADA-deficient SCID and correction of the phenotype associated with knockout of the *Ada gene*. Further studies were performed to understand issues surrounding scalability, species-specific vector systems, and the best approach for modeling biodistribution after systemic LV administration.[6,16] In the studies presented herein, we investigated age, dose, dosing schedules, re-administration, and the potential for viral inactivation after repeat administration in the ADA-deficient mouse model and in healthy young rhesus monkeys. Although the ADA-deficient mouse model was included to address specific questions about ADA deficiency and the potential of ectopic ADA expression supplied *in vivo* by LV-transduced tissues, the results presented have implications for other diseases in which LV-mediated gene transfer *in vivo* has shown promise.

To examine how age at treatment affects biodistribution, $Ada^{-/-}$ mice were treated as Neonates or as Adults previously maintained on ERT using PEG-ADA (as $Ada^{-/-}$ mice live no longer than 3 weeks without an exogenous source of ADA enzyme). $Ada^{-/-}$ Neonates were treated with a LV dose previously found to be necessary for survival. When treating the $Ada^{-/-}$ Adult mice, however, we were unable to deliver the concentrated LV dose within a single 100 μL injection volume, and thus the mice were treated with a reduced dose. Survival was high in all $Ada^{-/-}$ mice treated with ADA LV and 1 month of ERT, which was necessary to avoid loss from inadequate ADA expression. The biodistribution was most likely affected by dose because overall marking in mice treated as adults was reduced. The reduction in VCN in liver, lung, thymus, and brain, however, far exceeded the difference in dose.

In parallel studies, rhesus monkeys were treated at birth (study 1) or at 4 months of age (study 2) with one or two different doses and analyzed at approximately 1 year of age (12 months or 8 months post transfer, respectively). In each study, rhesus monkeys were treated with the maximum dose possible within a 1 mL injection volume or a dose one-tenth lower. LV marking in liver was dose-dependent with 10-fold differences in dose level and did not differ in rhesus monkeys treated at 4 months of age with one-third the dose administered to rhesus at birth. Furthermore, those animals injected at 4 months of age versus on the day of birth had detectable marking in spleen, lymph nodes, and bone marrow. Taken together, these results suggest it may be possible to achieve a similar biodistribution by treating infants at 4 months of age compared to birth with less vector and may represent an important step toward future clinical development of this treatment modality based on age.

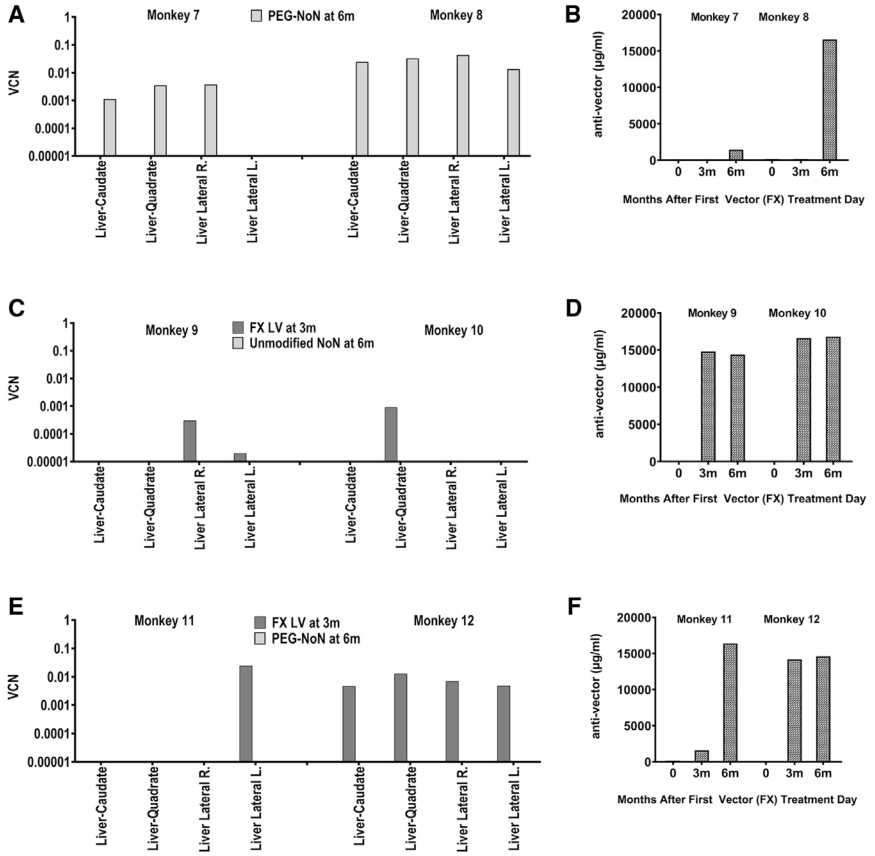

Figure 6. Re-Administration with a Pegylated LV Compared to an Unmodified LV in Immune Competent Rhesus Monkeys

Liver VCN (A, C, and E) and the concentration of anti-vector antibody detected in plasma (μg/mL) at 0 (pre), 3, and 6 months after the first vector treatment (B, D, and F) are shown for each pair of treated monkeys. (A and B) Rhesus monkeys #7 and #8 were administered the PEG-NeoNT LV at 6 months of age. (C and D) Rhesus monkeys #9 and #10 were administered the FX LV at 3 months of age and the unmodified NeoNT LV at 6 months of age. (E and F) Rhesus monkeys #11 and #12 were administered the FX LV at 3 months of age and the PEG NeoNT LV at 6 months of age.

Liver-directed therapy by systemic administration of LVs to treat disease states caused by monogenic mutations, such as ADA-deficiency, mucopolysaccharidoses (MPS: I, IIIa, VII), or hemophilia (Factor VIII or IX) may require re-administration of vector at some point after the initial treatment, because there is currently no evidence that cells with self-renewing capacity are transduced. Most of the adaptive immune responses reported after systemic LV administration were directed toward the transgene product expressed *in vivo*.[21,22] For example, one study found transgene-specific cytotoxic and humoral responses to Factor IX expressed in liver[2] and another described cytotoxic T cell responses specific to luciferase expressed in the lungs,[23] which resulted in reduced transgene expression. In our studies, we analyzed an anti-vector adaptive immune response and the potential for inactivation of subsequent LV administration in the ADA-deficient mouse model and in young rhesus monkeys. Furthermore, in the monkey studies, the use of non-expressed transgenes limited the potential for transgene-specific responses.

In single-exposure studies with unmodified ADA LV, neither immune-deficient $Ada^{-/-}$ mice nor immune competent $Ada^{+/-}$ mice treated as neonates had no or little anti-vector IgG response. This finding is more likely related to the tolerizing effect of neonatal exposure to vector, as has been demonstrated in studies with adeno-associated viral vectors (AAV).[24–26] In $Ada^{-/-}$ mice treated at 4 months of age (after 4 months of ERT), the concentration of anti-vector antibody increased 15,000- to 20,000-fold, a magnitude of increase not observed in immune-competent $Ada^{+/-}$ mice treated as adults. The extent of the response suggests a dysregulated response in $Ada^{-/-}$ mice treated as adults after treatment with PEG-ADA ERT. Indeed, dysregulation of the immune system after immune reconstitution with PEG-ADA ERT has been reported in $Ada^{-/-}$ mice and ADA-deficient patients.[27,28] Furthermore, responses in those $Ada^{-/-}$ mice treated with LV twice within 3 days had higher levels of IgG and diminished marking in liver and spleen. Others have shown that systemic administration of LV can also activate the innate immune response, resulting in the activation of Toll-like receptor (TLR)-dependent and -independent pathways[29] and higher numbers of transduced macrophages than transduced hepatocytes.[30] The double administration of LVs within 3 days may have enhanced the transduction of macrophages, ultimately resulting in a heightened adaptive response.

We also investigated the potential for pegylation of VSV-G LV to reduce immunogenicity. We hypothesized that a PEG-modified LV would prevent an adaptive immune response, evade pre-existing responses from an initial administration, and preserve transduced liver cells. Pegylation of viral vectors was demonstrated to increase stability and reduce immunogenicity in a variety of viral vector systems including LVs.[19] Systemic administration of PEG-modified VSV LV resulted in a longer half-life and increased marking in spleen, liver, and bone marrow of mice compared to unmodified VSV LV. To PEG-modify the LV, we developed a novel technique for conjugating PEG molecules to VSV during the concentration phase of vector production without a significant reduction in titer. Additionally, free unconjugated PEG molecules were successfully removed during concentration at the buffer exchange step of tandem flow filtration (TFF).[20] To compare the effects of pegylation, we split vector batches into two fractions, with one-half pegylated and the

other half mock-pegylated. Studies were carried out in immune-competent $Ada^{+/-}$ mice and healthy young rhesus monkeys.

The use of PEG-modified LV was safe as there were no adverse events in immune-competent $Ada^{+/-}$ mice when treated with PEG-modified LV as the sole treatment or as part of a dual treatment. $Ada^{+/-}$ mice treated as adults with a single administration of the ADA LV, the unmodified eGFP LV, or PEG-eGFP LV, also showed no differences in either the anti-vector IgG concentration or in liver VCN. However, when $Ada^{+/-}$ mice were treated with the PEG-eGFP LV once at 5 months and again at 8 months, the anti-vector response was significantly higher compared to those mice treated once with eGFP LV or PEG-eGFP, suggesting no protection with pegylated VSV LV.

Further, in re-administration studies with unmodified and PEG-modified LV over several months, both mice treated as adults and newborn or infant rhesus monkeys completely inactivated the second-administered LV resulting in no detectable marking. When mice were treated with either the unmodified eGFP LV or the PEG-modified eGFP LV at 5 months, followed by the ADA LV at 8 months, the ADA LV was not detected in the liver. When rhesus monkeys were treated with the non-expressed FX LV at 1 or 3 months of age (study 4) and then with either the unmodified or pegylated non-expressed NeoNT LV, the NeoNT LV was not detected in the liver. Because this finding was similar when comparing LVs carrying expressed transgenes or non-expressed transgenes, it is not likely that the transgene product was the neoantigen. In addition, these results suggest that the PEG-modified LV could not prevent inactivation. However, the concentration of anti-vector antibody detected when the first vector was PEG-modified was more than 10-fold lower than when the first vector was unmodified. These findings also suggest that the first exposure is sufficiently strong to elicit an adaptive immune response that may be attenuated with age. These findings are unlike those found with AAV serotype 8; when treatment was delayed from a newborn to 1 month of age, the antibody response to capsid was decreased.[31]

In an additional study, when mice were treated with LV three times, once as a neonate, once at 5 months, and again at 8 months of age, there was acute toxicity and 40% of the mice succumbed within 1 h of the third treatment. The same number of animals was lost when the third LV was unmodified LV or PEG-modified LV. Despite the acute toxicity, in the surviving mice the inactivation of the last LV was not complete, with the third vector detectable regardless of whether it was PEGylated or not. Indeed, in all $Ada^{+/-}$ mice treated as neonates, the unmodified ADA LV resulted in detection of subsequent vectors, suggesting neonatal administration may have induced tolerance and the potential for inactivation but was insufficient to prevent toxicity when the third treatment was with unmodified or pegylated LV. Furthermore, these results are in line with findings in rhesus monkeys where no adverse events were observed and suggest that PEG-modified vectors did not prevent inactivation or acute toxicity.

To characterize the anti-vector response, we performed cross-reactivity studies using different capture antigens (SIV LV versus HIV LV, LV pseudotyped with VSV-G versus ecotropic envelope versus amphotropic envelope, VSV-gamma retroviral vector (gRV) versus VSV-LV) with murine and rhesus monkey samples. The results of these comparisons demonstrated that the response was not transgene specific, vector backbone specific, capsid specific, or envelope specific. Furthermore, studies conducted using lysates from transfected HEK293T cells as capture antigen indicated that gag pol proteins or VSV-G were not responsible for the entire response observed because the overall responses were over 1,000-fold lower than those seen with packaged LV.

We hypothesized that the adaptive anti-vector response is polyclonal in nature, with antibodies generated to a variety of components in common with the various HIV-1 and SIV LV used in these studies, including other viral proteins and producer cell DNA and proteins. There are limited re-administration studies to support this hypothesis, but one study demonstrated that sera from rats injected with a VSV-G-eGFP LV into the striatum followed by a subcutaneous injection of the same LV or a different VSV-G-Factor IX LV resulted in antibodies to capsid protein, matrix protein, and VSV-G proteins as detected by immunoblot and that capsid-specific antibodies blocked subsequent LV administration.[32]

Other studies suggest that more than viral proteins can act as antigens. Proteomics analysis of LVs revealed an array of human packaging cell proteins, including nuclear proteins, elongation factors, chaperone proteins, and heat shock proteins, co-packaged into the virion and released upon entry into the cell.[33] It is not clear whether these proteins would elicit a response in humans, but they may be antigenic when administered to mice and nonhuman primates. In another study, it was demonstrated that VSV-G-pseudotyped LV produced by transient transfection contain tubulovesicular structures (TVSs) that are similar to micro-vesicles found in viral stocks of HIV-1, which were determined to contain producer cell DNA and proteins and residual plasmid DNA and proteins present in the cell culture medium.[34] These authors found that plasmid DNA associated with TVSs, but not VSV-G, was shown to elicit a Toll-like receptor 9 (TLR 9)-dependent response and was a potent adjuvant when co-administered with ovalbumin to produce T and B cell adaptive responses, concluding that TVS-associated plasmid may result in a similar finding with other viral proteins.

It is unclear whether systemic delivery of LV could be used as an additional treatment option for many ADA-deficient SCID patients, given the success with *ex vivo* gene therapy using gamma-retroviral or LV to genetically modified stem cells. Nevertheless, these studies are important for highlighting the complexities involved in developing novel therapeutics for SCID and other disorders. These studies reveal several factors that may be important to consider when translating liver-directed gene transfer with systemic administration of LVs for any indication. Specifically, we have demonstrated that treatment age, immune status, and frequency of administration

can impact outcomes. Lastly, additional safeguards should be considered, such as requiring that LV production occur in human cells to reduce immunogenicity and the development of specific screening methods to track immunological responses after systemic administration of LV, particularly before considering re-administration. Screening is routinely performed in nonhuman primate studies conducted with AAV to avoid using animals with pre-existing neutralizing antibodies.[31] Further strategies may need to be adapted from the AAV field, such as pre-treatment with immunosuppressive agents, particularly in immune-competent individuals.[35]

MATERIALS AND METHODS

Vectors

Vector maps are depicted in Figure S1. The HIV-1-based, self-inactivating (SIN) LV CSO-rc-MNDU3-ADA (MND-ADA LV), CCL-MNDU3-eGFP (eGFP LV), and the SIV-based LV CL20c-SM-hADA (SIV-ADA LV), CL20c-SM-NeoNT (NeoNT LV), and CL20c-SM-FXM10 (FX LV) were as previously described.[9,16] The EFS-ADA LV which contains the shortened version of the elongation factor 1 alpha gene promoter (EFS) was also described previously.[36] The EFS-ADA LV was modified to include the A2 ubiquitous chromatin opening element (UCOE, 1.5 fragment), upstream of the EFS promoter (UCOE-EFS-ADA LV).[37] The HIV-based and SIV-based vectors were packaged and titer was determined as previously reported.[16,20] Tissue VCN and ADA activity for each vector are shown in Figure S4.

Pegylation of Lentiviral Vectors

A 2-liter preparation of LV was prepared by transient transfection in 10% X-Vivo15 (Lonza, Allendale, NJ, USA) and partially concentrated (~70-fold) with the TFF Diafiltration System (Spectrum Laboratories, Rancho Dominguez, CA, USA).[20] The partial concentrate was diluted in Dulbecco's phosphate-buffered saline (DPBS) (Mediatech, Manassas, VA, USA) to a volume of 30 mL and was split into two fractions. The protein concentration of each fraction was determined (Pierce BCA Protein Assay, Pierce, Waltham, MA, USA). Succinimidyl-succinate activated monomethoxypoly (ethylene) glycol, mw-5000 (SSPEG, Sigma-Aldrich, St. Louis, MO, USA), was prepared as a 10× solution of 100 μg/μL in DPBS. Using the volume of the fraction to be pegylated and the total amount (μg) of vector protein pegylated to determine the appropriate volume of 10× SSPEG to add, 10 μg of SSPEG was used per μg of vector protein. Succinimidyl-succinate activated monomethoxypoly (ethylene) glycol, mw-5000 (SSPEG, Sigma-Aldrich, St. Louis, MO, USA), was prepared as a 10× solution of 100 μg/μL in DPBS. The 10× SSPEG was diluted with sufficient DPBS to match the volume of the vector to be pegylated and was added to the vector in a 1:1 ratio. To mock-pegylate the second fraction of vector, we diluted the vector 1:1 with DPBS. Both fractions were incubated for 1 h at 25°C with gentle agitation. To bind the free SSPEG molecules, we added L-lysine (Sigma, St. Louis, MO, USA) at 10 μg per μg of SSPEG added. Each fraction was returned to TFF to further concentrate the vector another ~40-fold, as well as remove the excess lysine, free SSPEG, and reaction byproducts. The PEG-modified LV and unmodifiedLV were stored as injectable aliquots at ≤−80°C. Titer was determined on HT-29 cells (ATCC HTB38) and the number of viral particles determined by p24 ELISA (Perkin Elmer, Waltham, MA, USA).[18]

Anti-PEG Direct Competitive ELISA

The concentration of PEG molecules was determined using a direct-competitive, high-sensitivity anti-PEG ELISA kit (Life Diagnostics, West Chester, PA, USA). Microtiter plates were pre-coated with PEG-BSA. The LV preparation was diluted 1:1,000 and applied to the microtiter plate. Horseradish peroxidase (HRP)-conjugated anti-mouse monoclonal antibody specific to the PEG backbone (50 μL) was added to the plate and incubated for 1 h with shaking. Conjugated and free PEG compete for the antibody such that the amount of antibody remaining was captured by the bound PEG-BSA. After washing the wells with PBS, 0.1 mL TMB (3,3′,5,5′-tetramethylbenzidine) was added to the wells and the plate was incubated for 20 min before stopping the reaction with 0.100 mL of 1N HCl. Absorbance was read at 450 nm. The amount of PEG in the sample was inversely correlated to the absorbance. The optical density (OD) of the samples was compared to a standard curve of diluted PEG-BSA to determine concentration of PEG (ng/mL) present in the vector preparations.

Mice

Mice were housed in accordance with the requirements of the Animal Welfare Act. All protocols adhere to NIH guidelines and were approved by the Animal Research Committee, Division of Laboratory Animal Medicine, at the University of California, Los Angeles (UCLA). The ADA-deficient mouse model was generated and rescued with a two-stage genetic engineering strategy previously described and characterized.[38] Due to the accumulation of adenosine and deoxyadenosine, the mice exhibit multi-system abnormalities, including the SCID phenotype prior to loss at postnatal day 19–20 (~3 weeks) from pulmonary insufficiency.[39] Mouse husbandry, genotyping procedures, and intravenous administration of LV were performed as previously described.[9] $Ada^{-/-}$ mice were maintained via weekly intramuscular injection of 50 U/kg body weight of pegylated bovine ADA (ADA-GEN; a kind gift from Leadiant Pharmaceuticals, Gaithersburg, MD, USA). All animals were housed in micro-insulator cages in a pathogen-free colony and all procedures were conducted in laminar flow hoods.

Mouse Studies

For systemic administration of LV into mice, vector aliquots were thawed quickly and diluted, if necessary, to a 50 or 100 μL injection volume in 0.9% saline (injection, USP; APP, Schaumburg, IL, USA) as described previously.[9] Entire litters of $Ada^{-/-}$ and $Ada^{+/-}$ neonates were injected with 50 μL of vector on postnatal day 1–3 via the superficial temporal vein using a 30G needle attached to a 1 mL syringe without sedation. Neonates received 2.5–5 × 10e10 TU/kg of an ADA LV (either MND-ADA LV, EFS-ADA LV, or UCOE-EFS-ADA LV). Approximately half of the litters treated were also given supplemental ERT for 30 days after LV injection.

Adult $Ada^{-/-}$ and $Ada^{+/-}$ mice were injected with 100 μL of vector via the tail vein with a 27G needle attached to a 1 mL syringe without sedation. Adult mice received 1–3.0 × 10e10 TU/kg of one of the ADA LV indicated above, and all $Ada^{-/-}$ adult mice continued to receive ERT for 1 month. Only $Ada^{+/-}$ adult mice received 1.5 × 10e10 TU/kg of PEG-eGFP LV or EGFP LV, and none received supplemental ERT (Table 1). Mice were analyzed at 4, 8, or 11 months after treatment for tissue VCN, immunophenotyping, and immune responses.

Rhesus Monkeys

All animal procedures conformed to the requirements of the Animal Welfare Act and protocols were approved prior to implementation by the Institutional Animal Care and Use Committee (IACUC) at the University of California, Davis. Normally cycling, adult female rhesus monkeys (*Macaca mulatta*) (N = 24) with a history of prior pregnancy were bred and identified as pregnant using established methods.[40] Activities related to animal care were performed according to California National Primate Research Center standard operating procedures. Newborns were delivered by cesarean section at term according to established protocols.[40] At birth, umbilical cord blood was collected.[41] Infants were raised in a dedicated nursery for postnatal studies with daily monitoring of physical signs and food intake. Weights were also monitored on a routine basis following established nursery protocols.

Monkey Study 1

Rhesus monkeys (Tables 1 and S1) were administered an intravenous injection of the SIV-ADA LV in ~1 mL volume, via a peripheral vessel at birth (n = 6; 2 females and 1 male [1 × 10e10 TU/kg], and 3 females [1 × 10e11 TU/kg]; ~500 g at birth). Blood samples (~1–3 mL) were collected from a peripheral vessel prior to vector administration, then at 1, 3, 7, 14, and 30 days, and then approximately every other month from 2 through 12 months of age (CBCs, chemistry panels, qPCR). Tissue harvests were performed at 12 months of age (12 months post-administration; body weight range at tissue harvest 1.8 kg to 2.5 kg).

Monkey Study 2

Rhesus monkeys (Tables 1 and S2) were administered an intravenous injection of the SIV-ADA LV in ~1 mL volume via a peripheral vessel at 4 months of age (n = 6; 2 females and 1 male [3.6 × 10e9 TU/kg] and 2 females and 1 male [3.6 × 10e10 TU/kg]; ~1 kg). Blood samples (~1–3 mL) were collected at 0, 1, 3, 7, 14, and 30 days, then approximately monthly from 2 through 10 months postnatal age (CBCs, chemistry panels, qPCR). Tissue harvests were performed at approximately 10 months of age (6 months post-administration; body weight range at tissue harvest 1.8 kg to 2.6 kg).

Monkey Study 3

Rhesus monkeys (n = 6, 2 per group; 3 females, 3 males; Table S2) were administered SIV LV carrying non-expressed transgenes (Table 1). They were treated with either the FX LV at 1 month of age or the NeoNT LV at 4 months of age only or with both the FX LV at 1 month of age and the NeoNT LV at 4 months of age. Blood samples (~1–3 mL) were collected at 0, 1, 3, 7, 14, and 30 days and then approximately monthly from 2 through 7 months of age (CBCs, chemistry panels, anti-vector ELISA, and qPCR). Tissue harvests were performed at 7 months of age (6 months post-vector administration when treated at 1 month of age or 3 months post-treatment when the last LV administration was at 4 months; body weight range at tissue harvest 1.7 kg to 2.1 kg).

Monkey Study 4

Rhesus monkeys (n = 6, 2 per group; 3 females, 3 males; Table S2) were administered SIV LV carrying non-expressed transgenes (Table 1). Monkeys were either treated with the FX LV at 3 months of age only, or with the FX LV at 3 months followed by the NeoNT LV at 6 months of age or the PEG NeoNT LV at 6 months of age. Blood samples (~1–3 mL) were collected at 0, 1, 3, 7, 14, and 30 days, then approximately monthly post-vector administration (CBCs, chemistry panels, anti-vector ELISA, and qPCR). Tissue harvests were performed at 10 months of age (4 months after the last vector administration; body weight range at tissue harvest 1.8 kg to 2.9 kg).

Post-Study Tissue Analysis

All animals were sedated with ketamine (10 mg/kg) then euthanized with an overdose of pentobarbital for tissue harvest according to established protocols.[41] Blood samples were collected, total body weights and measures were assessed, all organs were removed, and selected tissues weighed (e.g., brain, thymus, spleen, liver, adrenal glands, kidneys, and gonads). Tissues collected included the brain (cerebrum and cerebellum), lung (all lobes), trachea, esophagus, heart (right and left ventricle), aorta, pericardium, thymus, spleen, liver (all lobes), lymph nodes (right and left axillary and inguinal, tracheobronchial, and mesenteric), pancreas, right and left adrenal glands, right and left kidneys, reproductive tract (right and left gonads and uterus or right and left seminal vesicles and prostate), gastrointestinal tract (stomach, duodenum, jejunum, ileum, and colon), right and left muscular component of the diaphragm, omentum, right and left body wall (peritoneum), skin, muscle, and bone marrow. No abnormalities were detected during the in-life phase or at tissue harvest. All tissues collected for qPCR were placed in tubes and immediately frozen in liquid nitrogen and stored at ≤−80°C until assay.

VCN Quantification

VCN was determined by qPCR on genomic DNA extracted from cells and tissues. Primers and probes were previously described.[16] Quantitative real-time PCR analysis was carried out in 96-well optical plates using the 7900 ABI Sequence Detection System (Applied Biosystems, Foster City, CA, USA) and TaqMan Universal PCR Master Mix (Applied Biosystems, Foster City, CA, USA) according to the manufacturer's protocols and as previously described.[15] PCRs were run in triplicate in separate wells with 400 nM of each primer and 100 nM probe in a 25 μL reaction volume. The PCR protocol consisted of 1 cycle of 2 min at 50°C and 15 min at 95°C, followed by 40 cycles of 15 s at 95°C and 60 s at 60°C.

Anti-vector Indirect ELISA

The concentration of anti-vector IgG antibodies in plasma or sera was determined by ELISA. Concentrated VSV-G-pseudotyped LV (>1 × 10e8 TU/mL) was used as the capture antigen, and the entire procedure was performed to BSL2 standards in a biosafety cabinet with all excess vector, antibody, and washes aspirated into a 10% bleach solution. Nunc PVC 96 flat-well plates were coated (50 μL/well) with LV diluted 1:1,000 (~20 μg/mL) in 100 mM bicarbonate/carbonate binding buffer (3.03 g Na_2CO_3 and 6.0 g $NaHCO_3$ in 1,000 mL distilled water, pH 9.6) and incubated overnight at 4°C. The plates were washed three times with wash buffer (DPBS with 0.05% Tween-20; Sigma-Aldrich, St. Louis, MO, USA), followed by blocking with blocking buffer (DPBS with 1% fetal bovine serum [FBS]) for 1 h. A murine monoclonal anti-vector antibody at 200 μg/mL (sc-365019, Santa Cruz Biotechnology, Santa Cruz, CA, USA) was serially diluted 12× 1:10 (20 μg/mL) in blocking buffer for a total of 100 μL/well. Plasma samples were diluted 1:20 in blocking buffer and serially diluted 12× 1:10. The plate was incubated with gentle agitation at room temperature for 2 h and then washed 3× with wash buffer. Secondary goat anti-mouse IgG-HRP antibodies (sc-2005, Santa Cruz Biotechnology, Santa Cruz, CA, USA) were used to detect bound anti-vector mouse IgG, and goat anti-monkey IgG-HRP antibodies (sc-2458, Santa Cruz Biotechnology, Santa Cruz, CA, USA) were used to detect bound anti-vector monkey IgG. The secondary antibodies were diluted 1:2,000 in blocking buffer and 50 μL was added to each well. After 1 h incubation with gentle agitation, the wells were washed 3× with wash buffer. After aspiration of the last wash, 50 μL of HRP substrate (3,3′,5,5′-tetramethylbenzidine) liquid soluble HRP/chromatin/peroxidase substrate system (Moss, Pasadena, MD, USA) was added to each well. After a 5-min incubation at room temperature, 50 μL of HCl (0.1N) stop solution was added to each well. The absorbance (OD) was read at 450 nm and background subtracted out. The OD of the murine anti-vector monoclonal antibody dilutions were used to construct a standard curve using second-order log regression from which the experimental anti-vector IgG concentrations were determined.

Immunophenotypic Analysis

The lymphocyte immunophenotype was determined by flow cytometry, as previously described.[17] Briefly, cell suspensions were made from the large lobe of the mouse thymus and apical half of the mouse spleen using mechanical pressure through a 70 μm screen. Red blood cells were lysed, and the remaining cells were counted and stained. Thymocytes were double stained with anti-CD4 and anti-CD8 antibodies to determine the single positive and double positive/negative populations. Splenocytes were stained with anti-$CD45^+$, anti-$CD3^+$, and anti-$CD4^+$ or anti-CD3, anti-CD8, and anti-CD19. Gating on splenocyte subpopulations was as follows: $CD45^+$ followed by $CD3^+$ or $CD19^+$ or $CD3^+$ followed by $CD4^+$ or $CD8^+$. Absolute cell counts were normalized mathematically by multiplying the proportion of positive cells by the total cell count in the organ.

Statistical Analysis

Descriptive statistics such as number of observations, mean, and SEM were reported and presented in figures for quantitative measurements. For survival outcome, Kaplan-Meier method[42] and log-rank test[43] were used to summarize and compare the survival experience of mice across different groups. For quantitative outcome measurements, group-wise difference was assessed by nonparametric Wilcoxon rank sum test. The Dwass, Steel, Critchlow-Fligner Method[44] was used for p value adjustment due to multiple comparisons. The normality assumption was assessed before statistical testing. For all statistical investigations, tests for significance were two-tailed unless otherwise specified. A p value less than the 0.05 significance level was considered statistically significant. All statistical analyses were carried out using SAS version 9.3.[45]

SUPPLEMENTAL INFORMATION

Supplemental Information can be found online at https://doi.org/10.1016/j.omtm.2019.11.004.

AUTHOR CONTRIBUTIONS

Conceptualization: D.A.C.-S. and D.B.K.; Methodology: D.A.C.-S., A.F.T., and D.B.K; Investigation: D.A.C.-S., A.F.T., C.C.I.L., M.L.K., S.W., X.J., M.M., D.N.C., K.C., C.K., and C.L.H.; Statistical Analysis: X.W.; Writing, Original Draft: D.A.C.-S; Writing, Review & Editing: D.A.C.-S., A.F.T., D.B.K, C.L.H., D.N.C., and K.C.; Funding Acquisition: D.B.K. and A.F.T.; Supervision: D.A.C.-S., A.F.T., and D.B.K.

CONFLICTS OF INTEREST

D.A.C.-S. is an employee and shareholder of Orchard Therapeutics, a pharmaceutical company developing *ex vivo* HSC gene therapy products for the treatment of monogenic disorders. D.B.K. is a member of the Scientific Advisory Board at Orchard Therapeutics. Orchard has licensed a LV for gene therapy of ADA SCID from the University of California Regents on which D.B.K. is an inventor.

ACKNOWLEDGMENTS

These studies were supported by grants from the National Institute of Allergy and Infectious Diseases (NIAID, R01AI074043 to D.B.K.), the National Heart, Lung, and Blood Institute (NHLBI) Center for Fetal Monkey Gene Transfer for Heart, Lung, and Blood Diseases (HL085794 to A.F.T.), the Primate Center base operating grant (OD011107), and the Cores for Animal Care, Research Vectors, and Cell & Tissue Analysis from P01 HL073104 (to D.B.K.). The Flow Cytometry Core of the Eli and Edythe Broad Center for Regenerative Medicine & Stem Cell Research also supported these studies. Leadiant Pharmaceuticals (Gaithersburg, MD, USA) kindly provided ADA-GEN, and the authors thank Kristine David of Leadiant Pharmaceuticals for her helpful assistance.

REFERENCES

1. Naldini, L., Blömer, U., Gallay, P., Ory, D., Mulligan, R., Gage, F.H., Verma, I.M., and Trono, D. (1996). In vivo gene delivery and stable transduction of nondividing cells by a lentiviral vector. Science *272*, 263–267.

2. Follenzi, A., Battaglia, M., Lombardo, A., Annoni, A., Roncarolo, M.G., and Naldini, L. (2004). Targeting lentiviral vector expression to hepatocytes limits transgene-specific immune response and establishes long-term expression of human antihemophilic factor IX in mice. Blood *103*, 3700–3709.
3. Cantore, A., Ranzani, M., Bartholomae, C.C., Volpin, M., Valle, P.D., Sanvito, F., Sergi, L.S., Gallina, P., Benedicenti, F., Bellinger, D., et al. (2015). Liver-directed lentiviral gene therapy in a dog model of hemophilia B. Sci. Transl. Med. *7*, 277ra28.
4. Kobayashi, H., Carbonaro, D., Pepper, K., Petersen, D., Ge, S., Jackson, H., Shimada, H., Moats, R., and Kohn, D.B. (2005). Neonatal gene therapy of MPS I mice by intravenous injection of a lentiviral vector. Mol. Ther. *11*, 776–789.
5. McIntyre, C., Byers, S., and Anson, D.S. (2010). Correction of mucopolysaccharidosis type IIIA somatic and central nervous system pathology by lentiviral-mediated gene transfer. J. Gene Med. *12*, 717–728.
6. Di Natale, P., Di Domenico, C., Gargiulo, N., Castaldo, S., Gonzalez Y Reyero, E., Mithbaokar, P., De Felice, M., Follenzi, A., Naldini, L., and Villani, G.R. (2005). Treatment of the mouse model of mucopolysaccharidosis type IIIB with lentiviral-NAGLU vector. Biochem. J. *388*, 639–646.
7. van der Wegen, P., Louwen, R., Imam, A.M., Buijs-Offerman, R.M., Sinaasappel, M., Grosveld, F., and Scholte, B.J. (2006). Successful treatment of UGT1A1 deficiency in a rat model of Crigler-Najjar disease by intravenous administration of a liver-specific lentiviral vector. Mol. Ther. *13*, 374–381.
8. Kyosen, S.O., Iizuka, S., Kobayashi, H., Kimura, T., Fukuda, T., Shen, J., Shimada, Y., Ida, H., Eto, Y., and Ohashi, T. (2010). Neonatal gene transfer using lentiviral vector for murine Pompe disease: long-term expression and glycogen reduction. Gene Ther. *17*, 521–530.
9. Carbonaro, D.A., Jin, X., Petersen, D., Wang, X., Dorey, F., Kil, K.S., Aldrich, M., Blackburn, M.R., Kellems, R.E., and Kohn, D.B. (2006). In vivo transduction by intravenous injection of a lentiviral vector expressing human ADA into neonatal ADA gene knockout mice: a novel form of enzyme replacement therapy for ADA deficiency. Mol. Ther. *13*, 1110–1120.
10. Tarantal, A.F., and Skarlatos, S.I. (2012). Center for fetal monkey gene transfer for heart, lung, and blood diseases: an NHLBI resource for the gene therapy community. Hum. Gene Ther. *23*, 1130–1135.
11. Hirschhorn, R. (1990). Adenosine deaminase deficiency. Immunodefic. Rev. *2*, 175–198.
12. Hershfield, M.S. (1998). Adenosine deaminase deficiency: clinical expression, molecular basis, and therapy. Semin. Hematol. *35*, 291–298.
13. Chan, B., Wara, D., Bastian, J., Hershfield, M.S., Bohnsack, J., Azen, C.G., Parkman, R., Weinberg, K., and Kohn, D.B. (2005). Long-term efficacy of enzyme replacement therapy for adenosine deaminase (ADA)-deficient severe combined immunodeficiency (SCID). Clin. Immunol. *117*, 133–143.
14. Kohn, D.B., Hershfield, M.S., Puck, J.M., Aiuti, A., Blincoe, A., Gaspar, H.B., Notarangelo, L.D., and Grunebaum, E. (2019). Consensus approach for the management of severe combined immune deficiency caused by adenosine deaminase deficiency. J. Allergy Clin. Immunol. *143*, 852–863.
15. Xu, X., Tailor, C.S., and Grunebaum, E. (2017). Gene therapy for primary immune deficiencies: a Canadian perspective. Allergy Asthma Clin. Immunol. *13*, 14.
16. Carbonaro Sarracino, D., Tarantal, A.F., Lee, C.C., Martinez, M., Jin, X., Wang, X., Hardee, C.L., Geiger, S., Kahl, C.A., and Kohn, D.B. (2014). Effects of vector backbone and pseudotype on lentiviral vector-mediated gene transfer: studies in infant ADA-deficient mice and rhesus monkeys. Mol. Ther. *22*, 1803–1816.
17. Carbonaro, D.A., Jin, X., Cotoi, D., Mi, T., Yu, X.J., Skelton, D.C., Dorey, F., Kellems, R.E., Blackburn, M.R., and Kohn, D.B. (2008). Neonatal bone marrow transplantation of ADA-deficient SCID mice results in immunologic reconstitution despite low levels of engraftment and an absence of selective donor T lymphoid expansion. Blood *111*, 5745–5754.
18. Kweon, C.H., Kwon, B.J., Kim, I.J., Lee, S.Y., and Ko, Y.J. (2005). Development of monoclonal antibody-linked ELISA for sero-diagnosis of vesicular stomatitis virus (VSV-IN) using baculovirus expressed glycoprotein. J. Virol. Methods *130*, 7–14.
19. Croyle, M.A., Callahan, S.M., Auricchio, A., Schumer, G., Linse, K.D., Wilson, J.M., Brunner, L.J., and Kobinger, G.P. (2004). PEGylation of a vesicular stomatitis virus G pseudotyped lentivirus vector prevents inactivation in serum. J. Virol. *78*, 912–921.
20. Cooper, A.R., Patel, S., Senadheera, S., Plath, K., Kohn, D.B., and Hollis, R.P. (2011). Highly efficient large-scale lentiviral vector concentration by tandem tangential flow filtration. J. Virol. Methods *177*, 1–9.
21. Stein, C.S., Kang, Y., Sauter, S.L., Townsend, K., Staber, P., Derksen, T.A., Martins, I., Qian, J., Davidson, B.L., and McCray, P.B., Jr. (2001). In vivo treatment of hemophilia A and mucopolysaccharidosis type VII using nonprimate lentiviral vectors. Mol. Ther. *3*, 850–856.
22. Rowe, H.M., Lopes, L., Ikeda, Y., Bailey, R., Barde, I., Zenke, M., Chain, B.M., and Collins, M.K. (2006). Immunization with a lentiviral vector stimulates both CD4 and CD8 T cell responses to an ovalbumin transgene. Mol. Ther. *13*, 310–319.
23. Limberis, M.P., Vandenberghe, L.H., Zhang, L., Pickles, R.J., and Wilson, J.M. (2009). Transduction efficiencies of novel AAV vectors in mouse airway epithelium in vivo and human ciliated airway epithelium in vitro. Mol. Ther. *17*, 294–301.
24. Tai, D.S., Hu, C., Lee, C.C., Martinez, M., Cantero, G., Kim, E.H., Tarantal, A.F., and Lipshutz, G.S. (2015). Development of operational immunologic tolerance with neonatal gene transfer in nonhuman primates: preliminary studies. Gene Ther. *22*, 923–930.
25. Hinderer, C., Bell, P., Louboutin, J.P., Zhu, Y., Yu, H., Lin, G., Choa, R., Gurda, B.L., Bagel, J., O'Donnell, P., et al. (2015). Neonatal Systemic AAV Induces Tolerance to CNS Gene Therapy in MPS I Dogs and Nonhuman Primates. Mol. Ther. *23*, 1298–1307.
26. Tarantal, A.F., Lee, C.C.I., Martinez, M.L., Asokan, A., and Samulski, R.J. (2017). Systemic and Persistent Muscle Gene Expression in Rhesus Monkeys with a Liver De-Targeted Adeno-Associated Virus Vector. Hum. Gene Ther. *28*, 385–391.
27. Sauer, A.V., Brigida, I., Carriglio, N., and Aiuti, A. (2012). Autoimmune dysregulation and purine metabolism in adenosine deaminase deficiency. Front. Immunol. *3*, 265.
28. Sauer, A.V., Morbach, H., Brigida, I., Ng, Y.S., Aiuti, A., and Meffre, E. (2012). Defective B cell tolerance in adenosine deaminase deficiency is corrected by gene therapy. J. Clin. Invest. *122*, 2141–2152.
29. Brown, B.D., Sitia, G., Annoni, A., Hauben, E., Sergi, L.S., Zingale, A., Roncarolo, M.G., Guidotti, L.G., and Naldini, L. (2007). In vivo administration of lentiviral vectors triggers a type I interferon response that restricts hepatocyte gene transfer and promotes vector clearance. Blood *109*, 2797–2805.
30. Agudo, J., Ruzo, A., Kitur, K., Sachidanandam, R., Blander, J.M., and Brown, B.D. (2012). A TLR and non-TLR mediated innate response to lentiviruses restricts hepatocyte entry and can be ameliorated by pharmacological blockade. Mol. Ther. *20*, 2257–2267.
31. Wang, L., Bell, P., Lin, J., Calcedo, R., Tarantal, A.F., and Wilson, J.M. (2011). AAV8-mediated hepatic gene transfer in infant rhesus monkeys (Macaca mulatta). Mol. Ther. *19*, 2012–2020.
32. Abordo-Adesida, E., Follenzi, A., Barcia, C., Sciascia, S., Castro, M.G., Naldini, L., and Lowenstein, P.R. (2005). Stability of lentiviral vector-mediated transgene expression in the brain in the presence of systemic antivector immune responses. Hum. Gene Ther. *16*, 741–751.
33. Wheeler, J.X., Jones, C., Thorpe, R., and Zhao, Y. (2007). Proteomics analysis of cellular components in lentiviral vector production using Gel-LC-MS/MS. Proteomics Clin. Appl. *1*, 224–230.
34. Pichlmair, A., Diebold, S.S., Gschmeissner, S., Takeuchi, Y., Ikeda, Y., Collins, M.K., and Reis e Sousa, C. (2007). Tubulovesicular structures within vesicular stomatitis virus G protein-pseudotyped lentiviral vector preparations carry DNA and stimulate antiviral responses via Toll-like receptor 9. J. Virol. *81*, 539–547.
35. Corti, M., Cleaver, B., Clément, N., Conlon, T.J., Faris, K.J., Wang, G., Benson, J., Tarantal, A.F., Fuller, D., Herzog, R.W., and Byrne, B.J. (2015). Evaluation of Readministration of a Recombinant Adeno-Associated Virus Vector Expressing Acid Alpha-Glucosidase in Pompe Disease: Preclinical to Clinical Planning. Hum. Gene Ther. Clin. Dev. *26*, 185–193.
36. Carbonaro, D.A., Zhang, L., Jin, X., Montiel-Equihua, C., Geiger, S., Carmo, M., Cooper, A., Fairbanks, L., Kaufman, M.L., Sebire, N.J., et al. (2014). Preclinical demonstration of lentiviral vector-mediated correction of immunological and metabolic abnormalities in models of adenosine deaminase deficiency. Mol. Ther. *22*, 607–622.

37. Zhang, F., Frost, A.R., Blundell, M.P., Bales, O., Antoniou, M.N., and Thrasher, A.J. (2010). A ubiquitous chromatin opening element (UCOE) confers resistance to DNA methylation-mediated silencing of lentiviral vectors. Mol. Ther. *18*, 1640–1649.

38. Blackburn, M.R., Datta, S.K., and Kellems, R.E. (1998). Adenosine deaminase-deficient mice generated using a two-stage genetic engineering strategy exhibit a combined immunodeficiency. J. Biol. Chem. *273*, 5093–5100.

39. Blackburn, M.R., Datta, S.K., Wakamiya, M., Vartabedian, B.S., and Kellems, R.E. (1996). Metabolic and immunologic consequences of limited adenosine deaminase expression in mice. J. Biol. Chem. *271*, 15203–15210.

40. Tarantal, A. (2005). Ultrasound imaging in rhesus and long-tailed macaques: Reproductive and research applications. In The Laboratory Primate, S. Wolfe-Coote, ed. (Elsevier Academic Press), pp. 317–351.

41. Tarantal, A.F., McDonald, R.J., Jimenez, D.F., Lee, C.C., O'Shea, C.E., Leapley, A.C., Won, R.H., Plopper, C.G., Lutzko, C., and Kohn, D.B. (2005). Intrapulmonary and intramyocardial gene transfer in rhesus monkeys (Macaca mulatta): safety and efficiency of HIV-1-derived lentiviral vectors for fetal gene delivery. Mol. Ther. *12*, 87–98.

42. Kaplan, E.L., and Meier, P. (1958). Nonparametric-Estimation from Incomplete Observations. J. Am. Stat. Assoc. *53*, 457–481.

43. Mantel, N. (1966). Evaluation of survival data and two new rank order statistics arising in its consideration. Cancer Chemother. Rep. *50*, 163–170.

44. Fitzmaurice, G., Davidian, M., Verbeke, G., and Molenberghs, G. (2008). Longitudinal Data Analysis (Chapman and Hall/CRC).

45. SAS Institute (2012). SAS/STAT User's Guide, Version 9.3, 9.3 Ed. (SAS Institute).

OMTM, Volume *16*

Supplemental Information

Dosing and Re-Administration of Lentiviral Vector for *In Vivo* Gene Therapy in Rhesus Monkeys and ADA-Deficient Mice

Denise A. Carbonaro-Sarracino, Alice F. Tarantal, C. Chang I. Lee, Michael L. Kaufman, Stephen Wandro, Xiangyang Jin, Michele Martinez, Danielle N. Clark, Krista Chun, Colin Koziol, Cinnamon L. Hardee, Xiaoyan Wang, and Donald B. Kohn

Supplemental Figures and Tables

Figure S1. Vector Maps. All vectors are self-inactivating (SIN) lentiviral vectors (LV), with the U3, R, and U5 regions of the LTR shown with the SIN deletion indicated by the "X". HIV-1 or $SIV_{mac1A11}$ packaging region (Ψ), 5' portion of the gag gene (gag), rev-responsive element (rre), and central polypurine tract (cPPT) are indicated. **(a)** Vectors used for the murine studies. All are VSV-pseudotyped SIN HIV-1 (CCL or CSO) vectors with either the MNDU3 enhancer/promoter (MND-ADA) or the short version of the elongation factor-1 promoter (EFS-ADA) with or without a 5' A2 ubiquitous chromatin opening element (UCOE) driving human *Ada cDNA* or eGFP expression. **(b)** Vectors used for the rhesus monkey studies. All are VSV-pseudotyped SIN $SIV_{mac1A11}$ LV (CL20) with the MSCV viral enhancer/promoter. The SIV-ADA expresses human *Ada cDNA*. SIV-FX LV carries the non-expressed gene sequences derived from PhiX174 and the dominant-negative human RevM10 gene. SIV-NeoNT LV carries the bacterial transposon neomycin-resistance gene with the translational start codon eliminated (NeoNT).

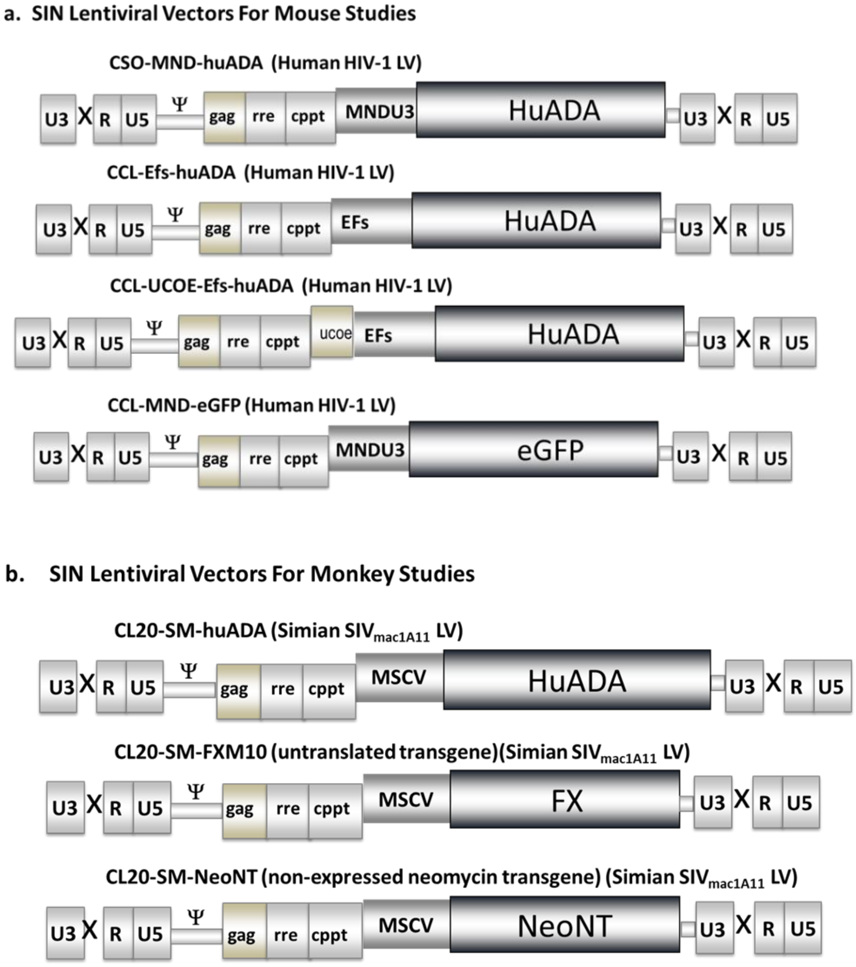

Figure S2: Immunophenotyping and absolute cell counts in mice systemically administered LV expressing human ADA. Immunophenotyping of cells by flow cytometry in thymus and spleen at time of analysis. *Ada+/-* mice untreated control (n= 9), *Ada-/-* mice treated with ERT only (n= 10), *Ada-/-* mice treated as adults (n=14), *Ada-/-* mice treated as adults twice within three days (n= 5), *Ada-/-* mice treated as neonates (n= 15), *Ada+/-* mice treated as neonates (n=13), *Ada-/-* mice untreated (16 days old) (n=6). **(a)** Absolute spleen and thymus cell counts (x10e7), total organ cell count (viable and non-viable). **(b-c)** Thymocyte subpopulations: single positive cells (SP CD4+ and SP CD8+), double positive cells (DP CD4+CD8+) and double negative cells (DN CD4-CD8-). (b) percentage in gate and (c) absolute counts (proportion x total organ cell count). **(d-e)** Splenocyte subsets: T cells (CD45+CD3+, CD3+CD4+, CD3+CD8+) and B cells (CD45+CD19+). **(d)** percentage in gate and **(e)** absolute counts (proportion x total organ cell count). Shown geometric means ±95% CI.

a.

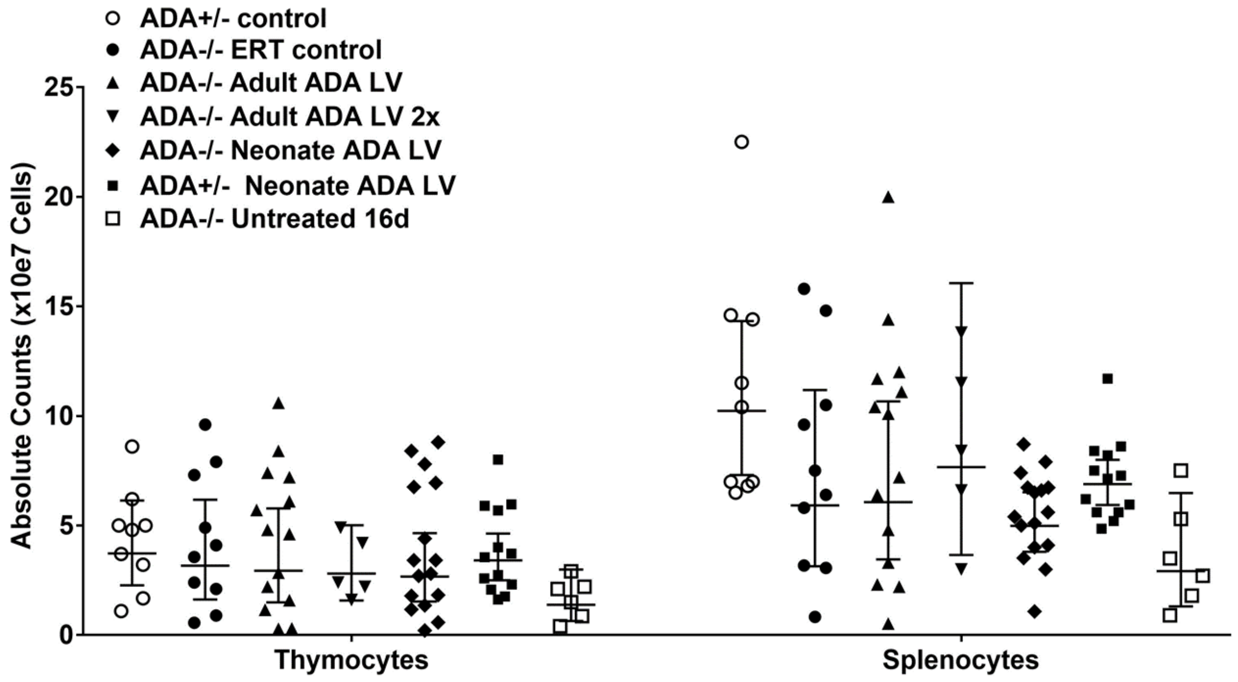

b.

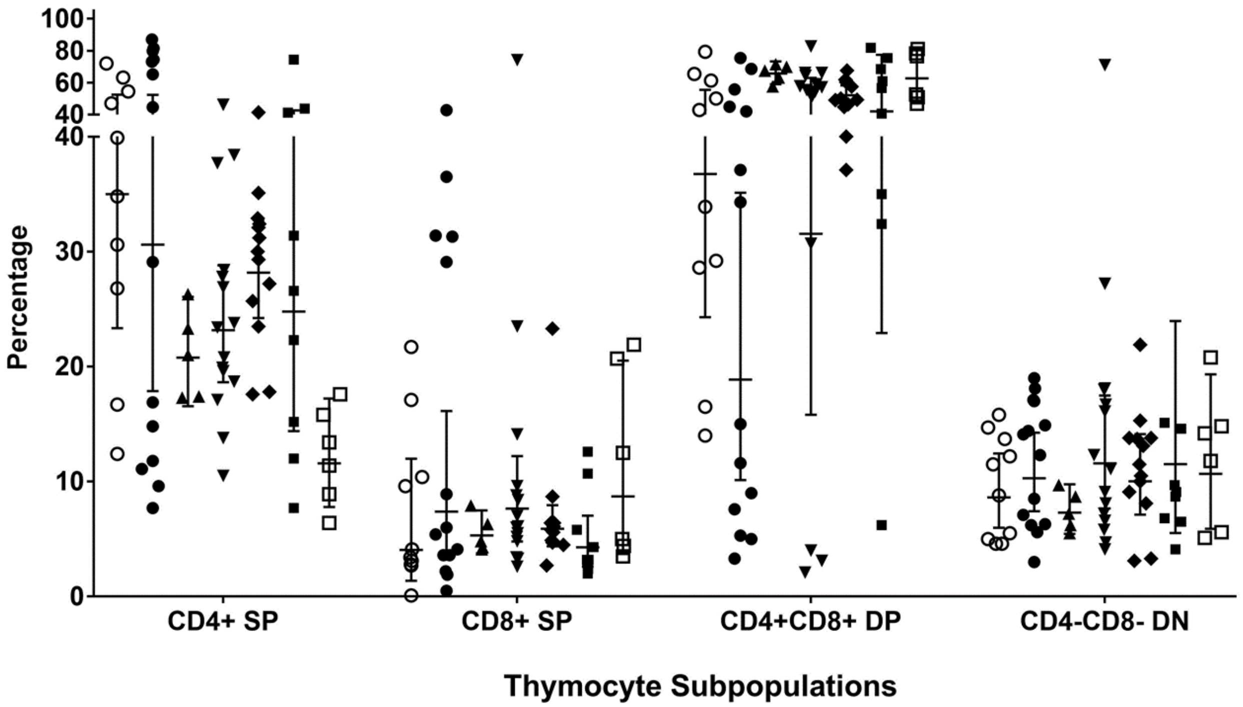

Percentage
100
80
60
40
40
30
20
10
0
CD4+ SP
CD8+ SP
CD4+CD8+ DP
CD4-CD8- DN
Thymocyte Subpopulations

c.

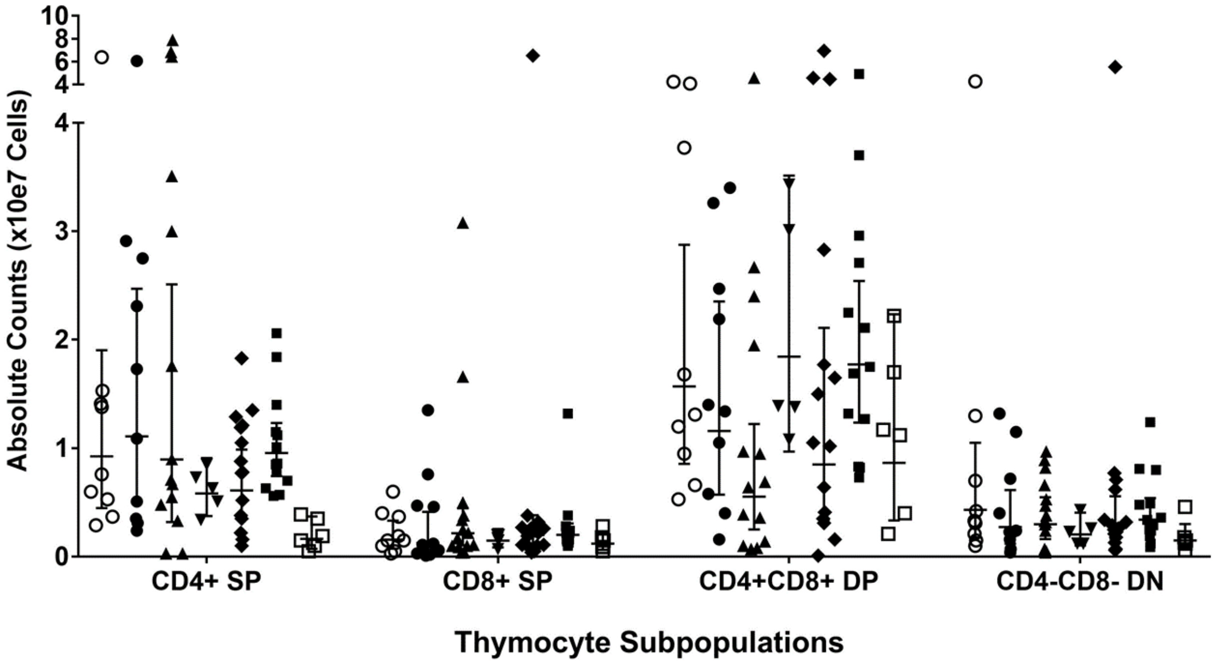

Absolute Counts (x10e7 Cells)
10
8
6
4
4
3
2
1
0
CD4+ SP
CD8+ SP
CD4+CD8+ DP
CD4-CD8- DN
Thymocyte Subpopulations

d.

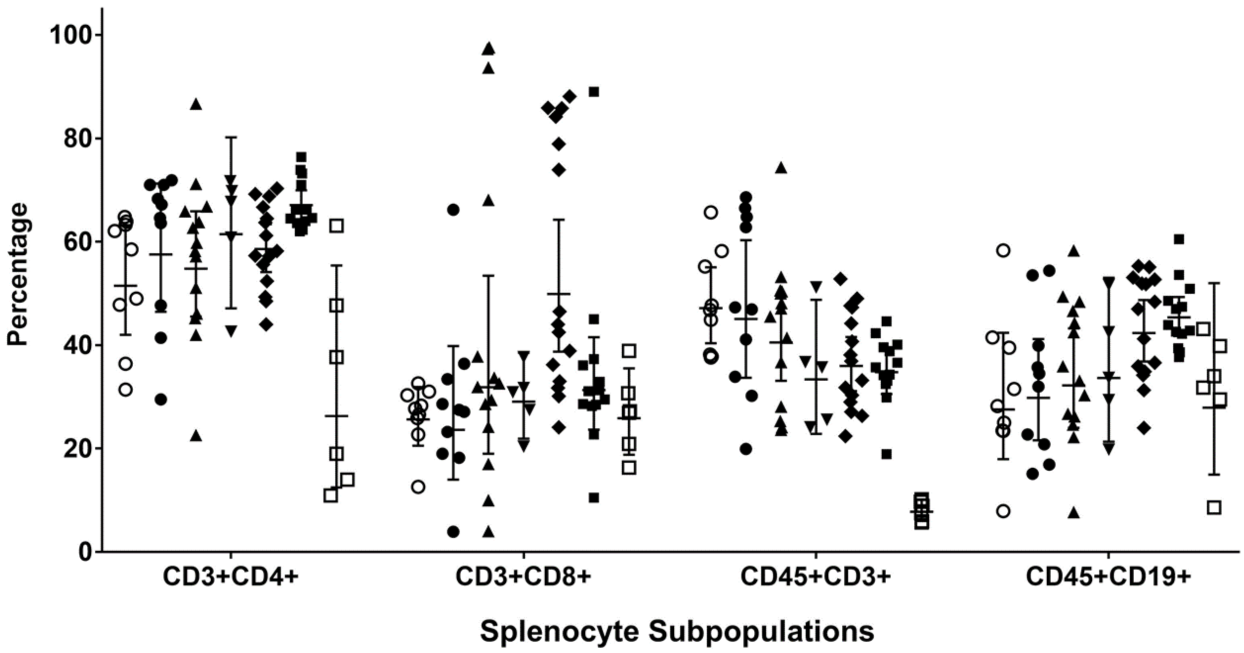

100
80
60
40
20
0
Percentage
CD3+CD4+
CD3+CD8+
CD45+CD3+
CD45+CD19+
Splenocyte Subpopulations

e.

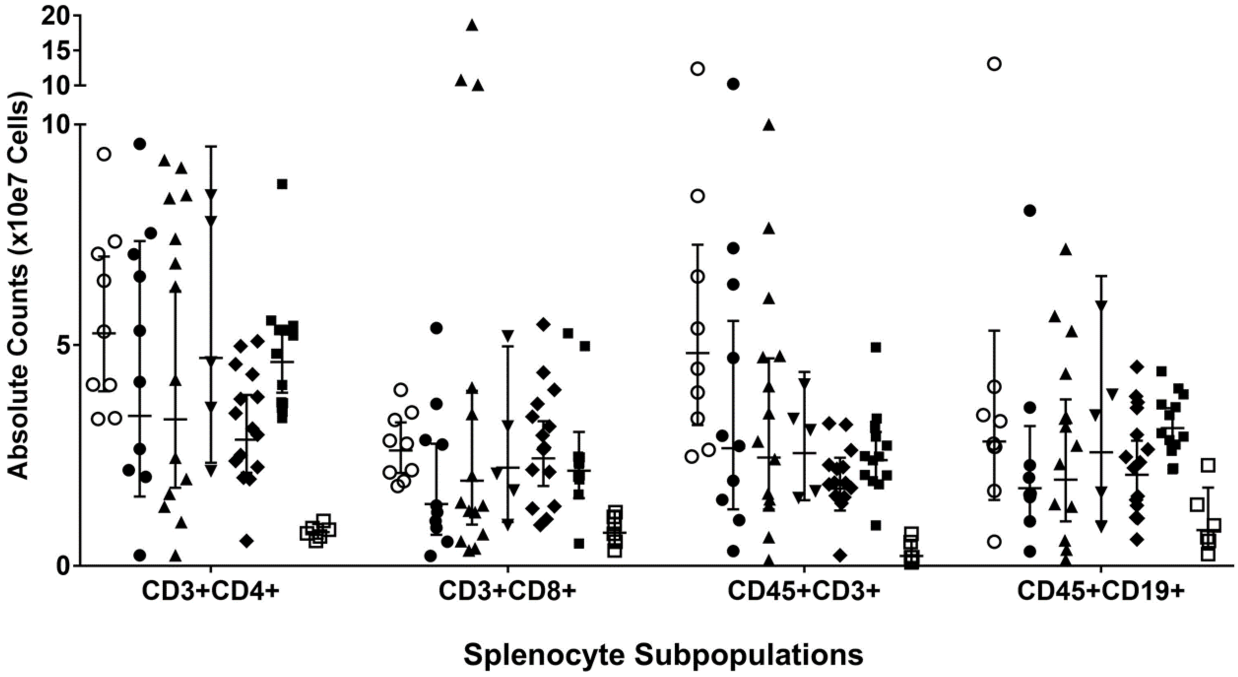

20
15
10
10
5
0
Absolute Counts (x10e7 Cells)
CD3+CD4+
CD3+CD8+
CD45+CD3+
CD45+CD19+
Splenocyte Subpopulations

Figure S3: Anti-vector response in HEK 293T lysates. ELISA using HEK 293T packaging cell line lysates (1:500) as the capture antigen compared to concentrated LV vector (1:1000 dilution). 293T cells were either transfected with the packaging plasmid 8.9 (expressing gag, pol, and rev), the env plasmid pHIT123 (VSV-G), or both.

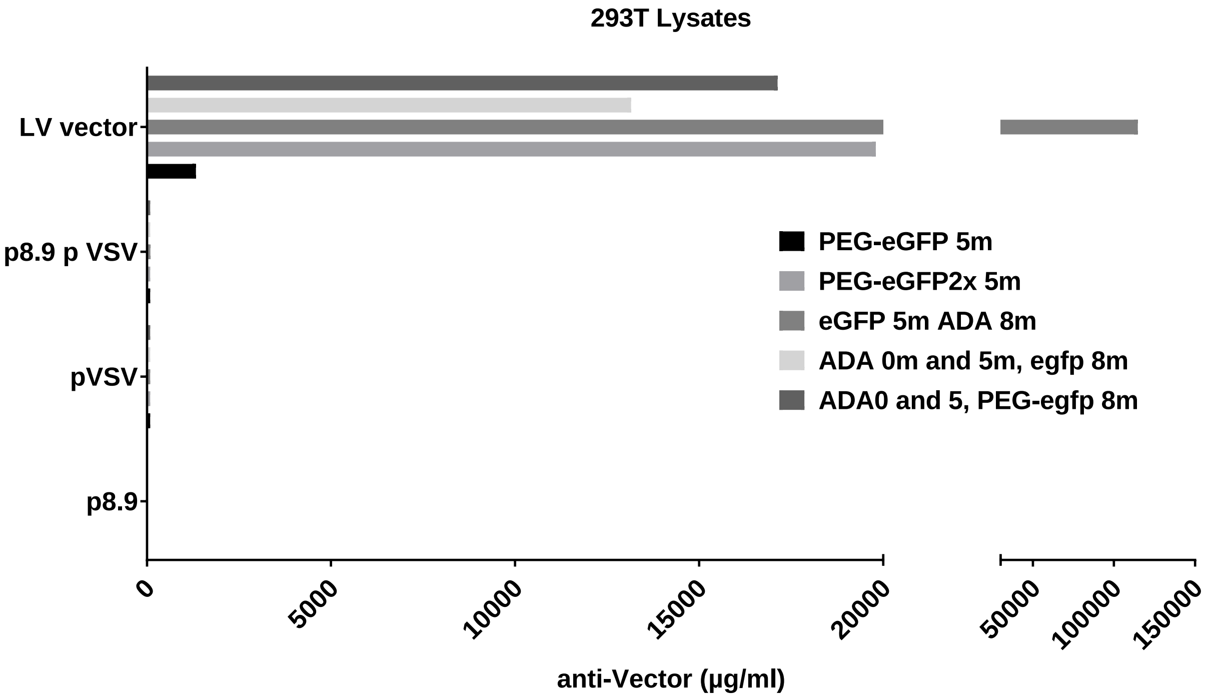

Figure S4. Spleen and liver VCN and ADA enzyme activity after systemic administration of MND-ADA LV, EFS-ADA LV or UCOE-EFS-ADA LV. Spleen (a-c) and Liver (d-f); (a and d) VCN was determined with ddPCR using primers and probes to human ADA as described previously (16); (b and e) ADA enzyme assay performed using the Diazyme ADA enzyme assay as described previously (16) (Unit=One unit of ADA is defined as the amount of ADA that generates one µmole of inosine from adenosine per mg per min at 37°C); (c and e) ADA Activity normalized for VCN (Units/VCN).

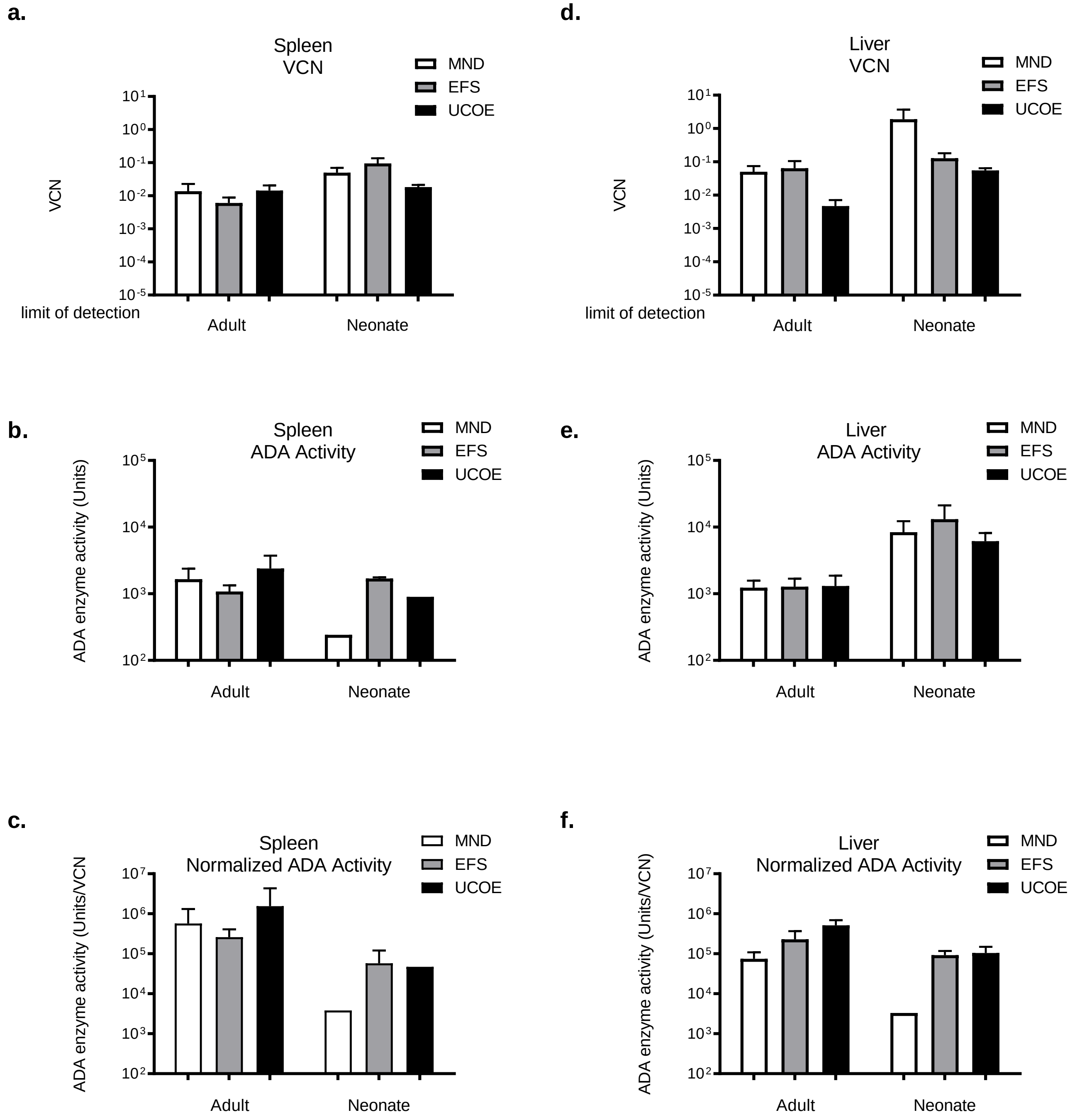

Table S1. Characterization of eGFP LV and PEG-eGFP LV

Vector Name	Fold Concentrated	PEG ELISA (ng/ml) 2500X dilution	PEG (ng/ml)	Titer (TU/ml)
Unconcentrated eGFP vector	0	0	0	2.1x10e6
Partially concentrated vector (PCV)	55	0	0	5.0x10e7
eGFP PCV	27.5	0	0	1.6x10e7
PEG-eGFP PCV	27.5	60	150,000	1.8x10e7
eGFP LV	1,000	0	0	6.6x10e8
PEG-eGFP LV	1,000	110	275,000	8.8x10e8

N/A=not available, PEG=polyethylene glycol, ELISA=enzyme linked immunosorbent assay

Table S2. Study design of rhesus monkey studies

Study number	Group (N)	Lentiviral Vectors (N)	Parameters
1<br>(n= 6)	Dose response at birth	SIV-ADA LV<br>1.0x10e10 TU/kg (3)<br>1.0x10e11 TU/kg (3)	Blood collection 0, 1, 3, 7, 14 and 30 days then every other month post-administration. Tissue harvest at 12 months of age.
2<br>(n= 6)	Dose response at 4 months	SIV-ADA LV<br>3.7x10e9 TU/kg (3)<br>3.7x10e10 TU/kg (3)	Blood collection 0, 1, 3, 7, 14 and 30 days then monthly post-administration. Tissue harvest at 10 months of age.
3<br>(n= 6)	Vector at 1 month<br>Vector at 4 months<br>Vector at 1 and 4 months	SIV-FX LF (2)<br>SIV-NeoNT LV (2)<br>SIV-FX LV+ SIV-NeoNT LV (2)	Blood collection 0, 1, 3, 7, 14 and 30 days then monthly post-administration. Tissue harvest at 7 months of age.
4<br>(n= 6)	Vector at 3 months<br>Vector at 6 months<br>Vector at 3 and 6 months	SIV-FX LV (2)<br>SIV-PEG-NeoNT LV (2)<br>SIV-FX LV+ SIV-PEG-NeoNT (2)	Blood collection 0, 1, 3, 7, 14 and 30 days then monthly post-administration. Tissue harvest at 10 months of age.

*All newborns were delivered by cesarean section and raised in the nursery for postnatal studies.
SIV=simian immunodeficiency virus ($SIV_{mac1A11}$ nonpathogenic clone); VSV=vesicular stomatitis virus glycoprotein; PEG=polyethylene glycol;
FX=Phi X-174 bacteriophage DNA; NeoNT=non-expressed neomycin resistance gene
**Tissue harvests included: brain (cerebrum, cerebellum), lung (all lobes), trachea, esophagus, heart, aorta, pericardium, thymus, spleen, liver (all lobes), lymph nodes (axillary, inguinal, tracheobronchial, mesenteric), pancreas, adrenals, kidneys, reproductive tract (gonads, uterus or seminal vesicles and prostate), gastrointestinal tract (stomach, duodenum, jejunum, ileum, colon), muscular component of the diaphragm, omentum, peritoneum (muscular component of body wall), skin, muscle, and bone marrow.

Table S3. Repeat Administration of SIV vectors: Liver marking and anti-vector response at study endpoint

Monkey Pairs	Vector at 1st time point	Vector at 2nd time point	Liver marking with FX-LV	Liver marking with NeoNT-LV	Baseline Anti-vector IgG	Anti-vector IgG 3 m after 1st time point	Anti-vector IgG 6 m after 1st time point
1 and 2	FX @ 1 m	N/A	Yes	N/A	No	Yes	Yes
3 and 4	N/A	NeoNT@ 4 m	N/A	Yes	No	No	Yes
5 and 6	FX @ 1 m	NeoNT @ 4 m	Yes	No	No	Yes	Yes
7 and 8	N/A	PEG-NeoNT @ 6 m	N/A	Yes	No	No	Yes
9 and 10	FX @ 3 m	NeoNT @ 6 m	Yes	No	No	Yes	Yes
11 and 12	FX @ 3 m	PEG-NeoNT @ 6 m	Yes	No	No	Yes	Yes

N/A=Not applicable, FX=non-expressed FXM10 gene, NeoNT=non-expressed neomycin resistance gene, m=month or months

Table S4. Characterization of the FX LV, NeoNT LV, and the PEG-NeoNT LV

Vector Name	Fold Concentrated	PEG ELISA (ng/ml) 5000X dilution	PEG (ng/ml)	Titer (TU/ml)
NeoNT unconcentrated vector	0	N/A	N/A	3.5x10e7
Partially concentrated vector	120	0	0	N/A
NeoNT PCV	26	0	0	N/A
PEG-NeoNT PCV	60	26	130,000	N/A
Flow through during concentration	N/A	21	105,000	N/A
FX unconcentrated vector	0	N/A	N/A	1.1x10e7
FX LV	1,000	N/A	N/A	2.4x10e9
NeoNT LV	1,000	0	0	1.6x10e10
PEG-NeoNT LV	1,000	28	140,000	9.6x10e9

N/A=not available, PEG=polyethylene glycol, PCV=partially concentrated vector, FX=non-expressed gene based on phi174, NeoNT=non-expressed neomycin resistance gene

Table S5. Response to 293T lysates (Mice)

			Bound Antigen<br>Transfected HEK 293T cell lysate<br>(1:500)			LV vector (1:1,000)
Time of Vector Administration			p8.9	pVSV	p8.9 p VSV	Avg anti-vector IgG response
0 months	5 months	8 months	µg/µL	µg/µL	µg/µL	µg/µL
	PEG-eGFP LV		7.8	90.0	89.0	1,330.0
	PEG-eGFP LV 2x		7.6	87.0	88.0	19,800.0
	eGFP-LV	ADA LV	8.0	88.0	91.0	114,666.7
ADA LV	ADA LV	eGFP LV	8.0	88.0	89.0	13,150.0
ADA LV	ADA LV	PEG-eGFP LV	7.6	87.0	88.0	17,133.3

Reactivity to lysates of 293T cells transfected with either the p8.9 (gag, pol, rev, tat), pHit123 (VSV-G), or both.
Lysates were used as bound antigen diluted 1:500 in carbonate binding buffer.
Concentrated LV was diluted 1:1000 in carbonate binding buffer.
Sera from treated *Ada+/-* mice, tested are pooled plasma n= 3-4 from each treatment arm.
